# Development of a novel AAK1 inhibitor via Kinobeads-based screening

**DOI:** 10.1038/s41598-024-57051-9

**Published:** 2024-03-20

**Authors:** Akari Yoshida, Satomi Ohtsuka, Fumiya Matsumoto, Tomoyuki Miyagawa, Rei Okino, Yumeya Ikeda, Natsume Tada, Akira Gotoh, Masaki Magari, Naoya Hatano, Ryo Morishita, Ayano Satoh, Yukinari Sunatsuki, Ulf J. Nilsson, Teruhiko Ishikawa, Hiroshi Tokumitsu

**Affiliations:** 1 Applied Cell Biology, Graduate School of Interdisciplinary Science and Engineering in Health Systems, Okayama University, Okayama, 700-8530 Japan; 2 Department of Science Education, Graduate School of Education, Okayama University, Okayama, 700-8530 Japan; 3 CellFree Sciences Co. Ltd, Matsuyama, 790-8577 Japan; 4 Organelle Systems Biotechnology, Graduate School of Interdisciplinary Science and Engineering in Health Systems, Okayama University, Okayama, 700-8530 Japan; 5 Graduate School of Natural Science and Technology, Okayama University, Okayama, 700-8530 Japan; 6 Department of Chemistry, Lund University, Box 124, 221 00 Lund, Sweden

**Keywords:** Biochemistry, Biotechnology, Chemical biology, Drug discovery

## Abstract

A chemical proteomics approach using Ca^2+^/calmodulin-dependent protein kinase kinase (CaMKK) inhibitor–immobilized sepharose (TIM-063-Kinobeads) identified main targets such as CaMKKα/1 and β/2, and potential off-target kinases, including AP2-associated protein kinase 1 (AAK1), as TIM-063 interactants. Because TIM-063 interacted with the AAK1 catalytic domain and inhibited its enzymatic activity moderately (IC_50_ = 8.51 µM), we attempted to identify potential AAK1 inhibitors from TIM-063-derivatives and found a novel AAK1 inhibitor, TIM-098a (11-amino-2-hydroxy-7*H*-benzo[de]benzo[4,5]imidazo[2,1-*a*]isoquinolin-7-one) which is more potent (IC_50_ = 0.24 µM) than TIM-063 without any inhibitory activity against CaMKK isoforms and a relative AAK1-selectivity among the Numb-associated kinases family. TIM-098a could inhibit AAK1 activity in transfected cultured cells (IC_50_ = 0.87 µM), indicating cell-membrane permeability of the compound. Overexpression of AAK1 in HeLa cells significantly reduced the number of early endosomes, which was blocked by treatment with 10 µM TIM-098a. These results indicate TIM-063-Kinobeads-based chemical proteomics is efficient for identifying off-target kinases and re-evaluating the kinase inhibitor (TIM-063), leading to the successful development of a novel inhibitory compound (TIM-098a) for AAK1, which could be a molecular probe for AAK1. TIM-098a may be a promising lead compound for a more potent, selective and therapeutically useful AAK1 inhibitor.

## Introduction

Protein kinases are enzymes encoded by over 500 genes in humans and play important signal transduction roles in cellular responses^1^. Protein kinase-mediated phosphorylation regulates many intracellular signaling pathways through functional changes in substrate proteins^2^. Congenital and acquired dysfunctions of protein kinases disrupt their roles in intracellular signal transduction mechanisms and cause many diseases, including cancer^3^. Therefore, many protein kinase inhibitors have been developed to treat diseases associated with the disruption of intracellular signaling mechanisms mediated by protein kinases, especially those based on excessive activation or overexpression of enzymes^4,5^. To date, 80 small-molecule protein kinase inhibitors have been approved by the US Food and Drug Administration (https://brimr.org/protein-kinase-inhibitors/: accessed January 29, 2024). Protein kinases utilize ATP as a phosphate donor; therefore, most protein kinase inhibitors suppress the catalytic activity of the enzyme in an ATP-competitive manner^6,7^. This often makes it difficult for protein kinase inhibitors to have strict specificity for target kinases because of the relatively similarity of their catalytic domain structure to that of other protein kinases^8^. Therefore, the presence of off-target kinases may have adverse effects. However, the fact that a single protein kinase inhibitor suppresses the activities of multiple off-target protein kinases may allow the development of off-target kinase inhibitors by modifying the inhibitor as a lead compound. We recently prepared Kinobeads^9,10^ in which a Ca^2+^/calmodulin-dependent protein kinase kinase (CaMKK) inhibitor (TIM-063, 2-hydroxy-3-nitro-7*H*-benzo[de]benzo[4, 5] -imidazo[2,1-*a*]isoquinolin-7-one) was immobilized on sepharose beads to characterize the interaction between TIM-063 and CaMKK^11,12^. In this study, we searched for TIM-063—interacting protein kinases using the Kinobeads technology^9,10^ and successfully identified candidates of multiple off-target protein kinases for TIM-063, including AP2-associated protein kinase 1 (AAK1), as well as the main targets, CaMKK isoforms. AAK1 is a member of the Numb-associated kinases (NAKs) family^13^ which also includes cyclin G-associated kinase (GAK)^14^, bone morphogenetic protein 2-inducible kinase (BIKE)^15^, and serine/threonine kinase 16 (STK16)^16^. AAK1 phosphorylates Thr156 in the µ subunit of the adaptor protein 2 (AP2)^17^ and Thr102 of Numb^18^ that plays important roles in clathrin-mediated endocytosis. AAK1 is involved in various neuronal disorders including schizophrenia^19^, Parkinson’s disease^20^, neuropathic pain^21^, amyotrophic lateral sclerosis (ALS)^22^, and Alzheimer’s disease^23^. In addition, AAK1 is involved in viral infections, including hepatitis C virus, dengue virus, Ebola virus^24,25^, and SARS-CoV-2^26^. Therefore, AAK1 is a potential therapeutic drug target^27,28^ and screening of clinically used kinase inhibitors via a thermal shift assay revealed multiple potential AAK1 inhibitors, including momelotinib (N-(Cyanomethyl)-4-(2-{[4-(morpholin-4-yl)phenyl]amino}pyrimidin-4-yl)benzamid), lestaurtinib ((5S,6S,8R)-6-hydroxy-6-(hydroxymethyl)-5-methyl-7,8,14,15-tetrahydro-*5H*-16-oxa-4b,8a,14-triaza-5,8-methanodibenzo[b,h]cycloocta[jkl]cyclopenta[e]-as-indacen-13(6*H*)-one), nintedanib (methyl (3Z)-3-{[(4-{methyl[(4-methylpiperazin-1-yl)acetyl]amino}phenyl)amino](phenyl)methylidene}-2-oxo-2,3-dihydro-1*H*-indole-6-carboxylate), fedratinib (N-tert-butyl-3-[(5-methyl-2-{[4-(2-pyrrolidin-1-ylethoxy)phenyl]amino}pyrimidin-4-yl)amino]benzenesulfonamide), sunitinib (*N*-[2-(diethylamino)ethyl]-5-[(*Z*)-(5-fluoro-1,2-dihydro-2-oxo-*3H*-indol-3-ylidine)methyl]-2,4-dimethyl-*1H*-pyrrole-3-carboxamide), baricitinib ({1-(ethylsulfonyl)-3-[4-(*7H*pyrrolo[2,3-*d*]pyrimidin-4-yl)-1*H*-pyrazol-1-yl]azetidin-3-yl}acetonitrile), and AZD-7762 (3-(carbamoylamino)-5-(3-fluorophenyl)-*N*-[(3*S*)-3-piperidyl]thiophene-2-carboxamide)^13^. Recently, a small-molecule chemical probe (SGC-AAK1-1, *N*-(6-(3-(*N*,*N*-diethylsulfamoyl)amino)phenyl)-1*H*-indazol-3-yl)cyclopropanecarboxamide), which is potent and selective for AAK1/BMP2K, was developed^29^ and used to evaluate the function of AAK1, including the promotion of clathrin-mediated endocytosis^30^. In the present study, we succeeded in developing a novel TIM-063 derived AAK1 inhibitor, TIM-098a, which did not inhibit CaMKK activity. We also examined the inhibitory effect of TIM-098a on AAK1 activity and AAK1-regulated endocytosis.

## Results and discussion

### Identification of TIM-063–interacting protein kinases using Kinobeads technology

**Figure 1:**
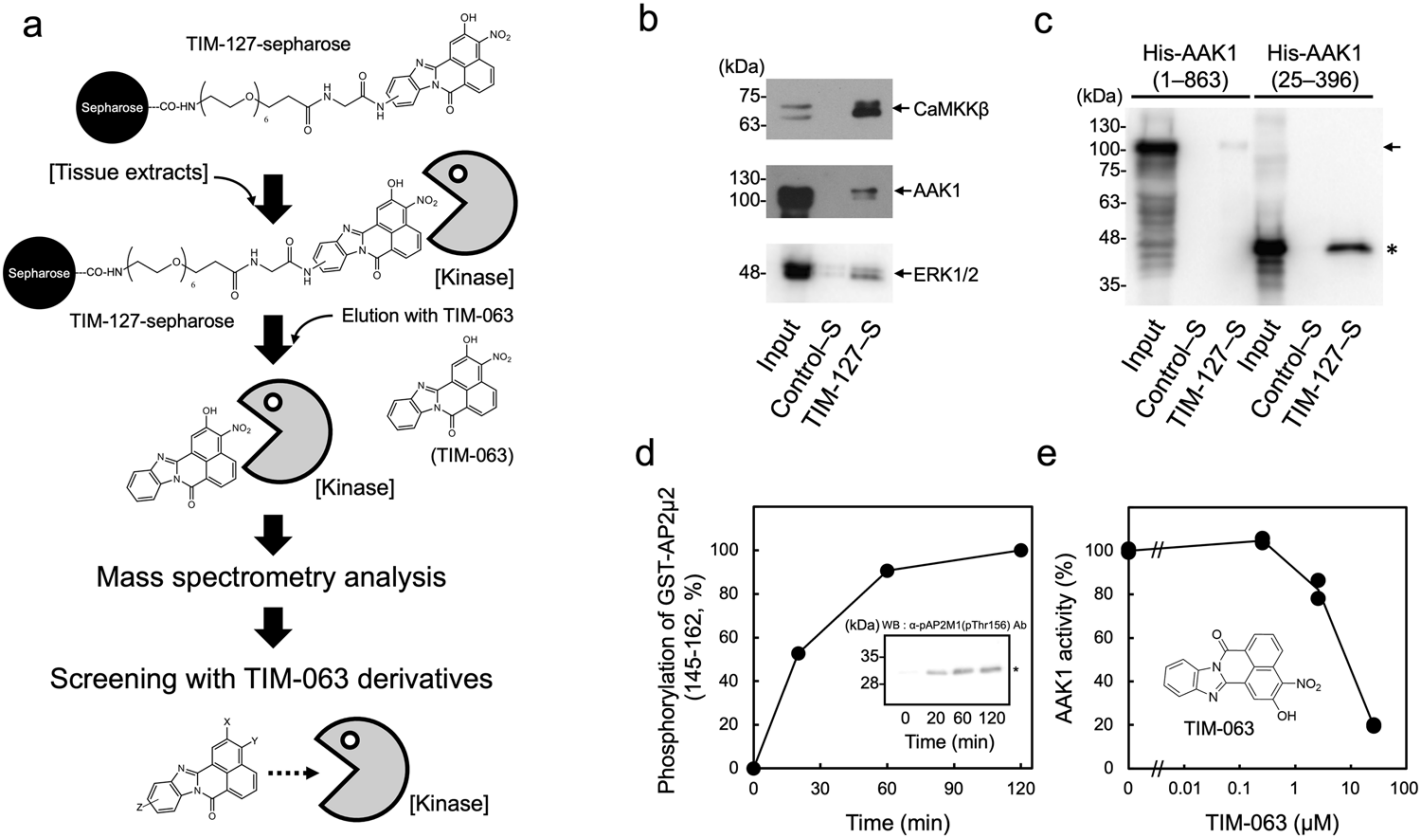
Identification and characterization of AAK1 as a TIM-063–interactant. (**a**) Protocol for identification of TIM-063–interactants using TIM-063–immobilized sepharose (TIM-127-sepharose^12^) combined with mass spectrometry analysis. Mouse cerebrum extracts were applied onto TIM-127-sepharose, and after extensive washing of resin, TIM-063–interactants were eluted by the addition of 100 µM TIM-063 containing buffer, followed by identification via mass spectrometry analysis as described in the “Methods”. (**b**) Eluates from TIM-127-sepharose (TIM-127–S) or a control sepharose without the inhibitor (Control–S) as described in (**a**) were analyzed by immunoblotting with either anti-CaMKKβ/2 antibody (*upper panel*), anti-AAK1 antibody (*middle panel*), or anti-ERK1/2 antibody (*bottom panel*) together with mouse cerebrum extracts (Input). (**c**) Extracts from COS-7 cells expressing His-tagged AAK1 wild type (1–863) or His-tagged AAK1 catalytic domain (25–396) were incubated with either TIM-127-sepharose or control sepharose, and after extensive washing of resin, TIM-063–interactants were eluted by the addition of 100 µM TIM-063–containing buffer, followed by immunoblot analysis with an anti-AAK1 antibody as described in the “Methods”. An arrow and asterisk indicate His-tagged AAK1 wild type (1–863) and His-tagged AAK1 catalytic domain (25–396), respectively. (**d**) Phosphorylation of GST-AP2µ2 (145–162) at Thr156 by the His-tagged AAK1 catalytic domain (25–396) for various time periods (0–120 min) was detected by immunoblot analysis with an anti-phospho-AP2µ2 (pThr156) antibody (*insert*), quantitated by densitometric scanning of the immunoreactive bands, and expressed as a percentage of the value at 120 min of the reaction. An asterisk indicates phospho-Thr156 in GST-AP2µ2 (145–162). (**e**) Inhibition of AAK1 activity by TIM-063 in a dose-dependent manner. Phosphorylation of GST-AP2µ2 (145–162) by His-tagged AAK1 catalytic domain (25–396) was measured in the presence or absence of various concentrations of TIM-063 at 30 °C for 20 min using 100 µM [γ-^32^P]ATP as described in the “Methods”. AAK1 activities are expressed as a percentage of the average value in the absence of the compound. Results represent duplicate experiments. Chemical structure of TIM-063 is indicated (*insert*). Molecular mass markers (kDa) are indicated in the left lanes of immunoblot panels.

TIM-063 (2-hydroxy-3-nitro-7*H*-benzo[de]benzo[4, 5]-imidazo[2,1-*a*]isoquinolin-7-one, Fig. 1a) was originally developed as a CaMKK inhibitor via screening of an STO-609–derived chemical library^11^ and TIM-063–immobilized sepharose (TIM-127-sepharose) was used to characterize the Ca^2+^/CaM-dependent interaction of CaMKK and the inhibitor^12^. Because TIM-063 is an ATP-competitive inhibitor similar to STO-609^31^, the inhibitor may interact and inhibit off-target kinases. If that is the case, TIM-063 could be a lead compound for development of inhibitors of the off-target kinases. Therefore, we attempted to identify TIM-063–interacting kinases from tissue extracts using TIM-127-sepharose combined with mass spectrometry analyses. Mouse cerebral extracts were incubated with TIM-127-sepharose followed by extensive washing of resin. Subsequently, TIM-063–interacting proteins were eluted by the addition of 100 µM TIM-063–containing solution, and then identified via mass spectrometry analysis (Fig. 1a). Consistent with results of a previous report^12^, CaMKKα/1 and β/2 were readily identified as the main targets of TIM-063 and two potential off-target protein kinases, AAK1 and ERK2, were detected by the proteomic approach (Table 1 and Supplemental Information S1). This was confirmed by immunoblot analyses with the corresponding antibodies, indicating that the protein kinases interacted with the TIM-063 motif on the inhibitor-immobilized sepharose but not with the control sepharose (Fig. 1b).

**Table 1:** TIM-063–binding protein kinases from mouse cerebrum^a^.

Protein ID	Molecular mass (Da)	Protein name	No. of peptides (% coverage)
KKCC2_MOUSE	64,576	Calcium/calmodulin-dependent protein kinase kinase 2 (CaMKKβ/2)	10 (18)
AAK1_MOUSE	103,282	AP2-associated protein kinase 1 (AAK1)	9 (11)
KKCC1_MOUSE	55,802	Calcium/calmodulin-dependent protein kinase kinase 1 (CaMKKα/1)	3 (5)
MK01_MOUSE	41,249	Mitogen-activated protein kinase 1 (ERK2)	2 (5)

^a^Mouse cerebrum protein kinases bound to TIM-063–immobilized sepharose were eluted and separated via SDS-PAGE. Slices were then cut from the gel, digested with trypsin, and identified using LC–MS/MS, as described in the “Methods”. The right column shows the number of identified peptides with sequence coverage (%) within parentheses. The identified peptides and their positions are listed in Supplemental Information S1.

### TIM-063 interaction and inhibition of AAK1

AAK1 was chosen for further investigation because AAK1 has been demonstrated to be a promising therapeutic target for schizophrenia^19^, Parkinson’s disease^20^, neuropathic pain^21^, Alzheimer’s disease^23^, and various viral infections^24–26^. First, to confirm the interaction of TIM-063 with AAK1, we performed a pull-down experiment on COS-7 cell extracts expressing His-tagged AAK1 using TIM-063–immobilized sepharose (TIM-127-sepharose) (Fig. 1c). In contrast to the weak binding of His-tagged AAK1 (full length, 1–863) with TIM-063, the catalytic domain of AAK1 (His-AAK 25–396) exhibited a strong interaction with TIM-063–immobilized sepharose (TIM-127-sepharose), but not with a control sepharose without an immobilized compound. These results suggest that TIM-063 interacts with the catalytic domain of AAK1, which might be partially autoinhibited by its interaction with its C-terminal domain; therefore, the TIM-063 interaction of full-length AAK1 is apparently weaker than that of the catalytic domain of AAK1.

AAK1 phosphorylates AP2µ2 at Thr156; therefore, we set up an AAK1 activity assay using GST-fused AP2µ2 fragment (residues 145–162) as a substrate and anti-phospho-Thr156 antibody for detection of the substrate phosphorylation by AAK1. The time course experiment showed that purified His-AAK 25–396 phosphorylates GST-AP2µ2 (145–162) at Thr156 (Fig. 1d); further AAK1 activity assays were performed with a reaction time of 20 min. When we tested the effect of TIM-063 on AAK1 activity using GST-AP2µ2 (145–162) and 0.1 mM [γ-^32^P]ATP with various concentrations of TIM-063, the compound could suppress the AAK1 activity in a dose-dependent manner with an IC_50_ value of 8.51 µM. These results suggest that the identification of the inhibitor–interacting kinase (AAK1) using the TIM-063-Kinobeads method reflects the inhibition of the kinase by the inhibitor utilized for the Kinobeads^9^.

### Screening for AAK1 inhibitors from the TIM-063-derivative chemical library

**Figure 2:**
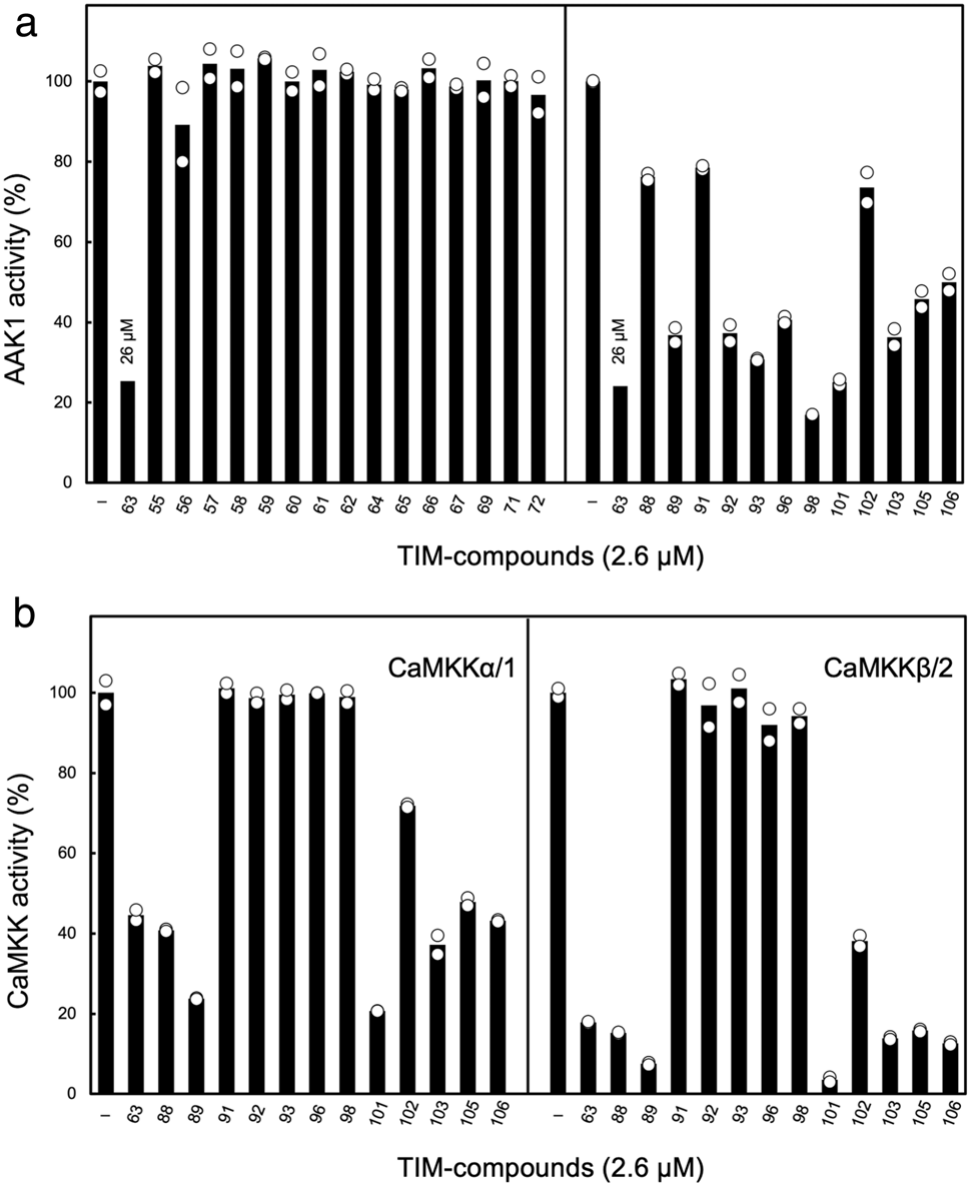
Characterization of TIM-063 derivatives as AAK1 inhibitors. (**a**) Protein kinase activities of His-tagged AAK1 catalytic domain (25–396) were measured at 30 °C for 20 min using 100 μM [γ-^32^P]ATP in the presence of 26 µM TIM-063 or 2.6 µM of its derivatives (TIM-055 − TIM-106) or absence as described in the “Methods”. AAK1 activities are quantitated and expressed as a percentage of the average value in the absence of the compound (–). Results represent duplicate experiments. (**b**) Protein kinase activities of CaMKKα/1 (*left panel*) and β/2 (*right panel*) were measured at 30 °C for 20 min using 100 µM [γ-^32^P]ATP, and 2 mM CaCl_2_ with 6 µM CaM in the presence of 2.6 µM TIM-063 or its derivatives (TIM-088 − TIM-106) or absence as described in the “Methods”. CaMKK activities are quantitated and expressed as a percentage of the average value in the absence of the compound (−). Results represent duplicate experiments.

The inhibitory potency of TIM-063 against AAK1 activity (IC_50_ = 8.51 µM, Fig. 1e) was moderate and approximately one-order lower than that against CaMKK isoforms (IC_50_ for CaMKKα/1 = 0.63 µM and IC_50_ for CaMKKβ/2 = 0.96 µM)^11^, indicating that TIM-063 is not a potent AAK1 inhibitor but can be used as a lead compound for developing potential AAK1 inhibitors. Therefore, we attempted to screen more potent AAK1 inhibitors than TIM-063 from the chemical library of TIM-063-derivatives (Fig. 2a, Table 2 and see in “Methods”). We used a compound concentration of 2.6 µM for the initial AAK1 inhibitor screening because 2.6 µM and 26 µM TIM-063 inhibits ~ 20% and ~ 80% of AAK1 activity, respectively (Fig. 1e). We also tested the effect of the compounds on the activities of CaMKK isoforms (α/1 and β/2) (Fig. 2b). Among the TIM-063-derivatives, TIM-098, which contains an amino group at the R_4_ position, was found to be the most potent AAK1 inhibitor, with an inhibitory potency > tenfold higher than that of TIM-063, without any effects on CaMKK activity.

**Table 2:**
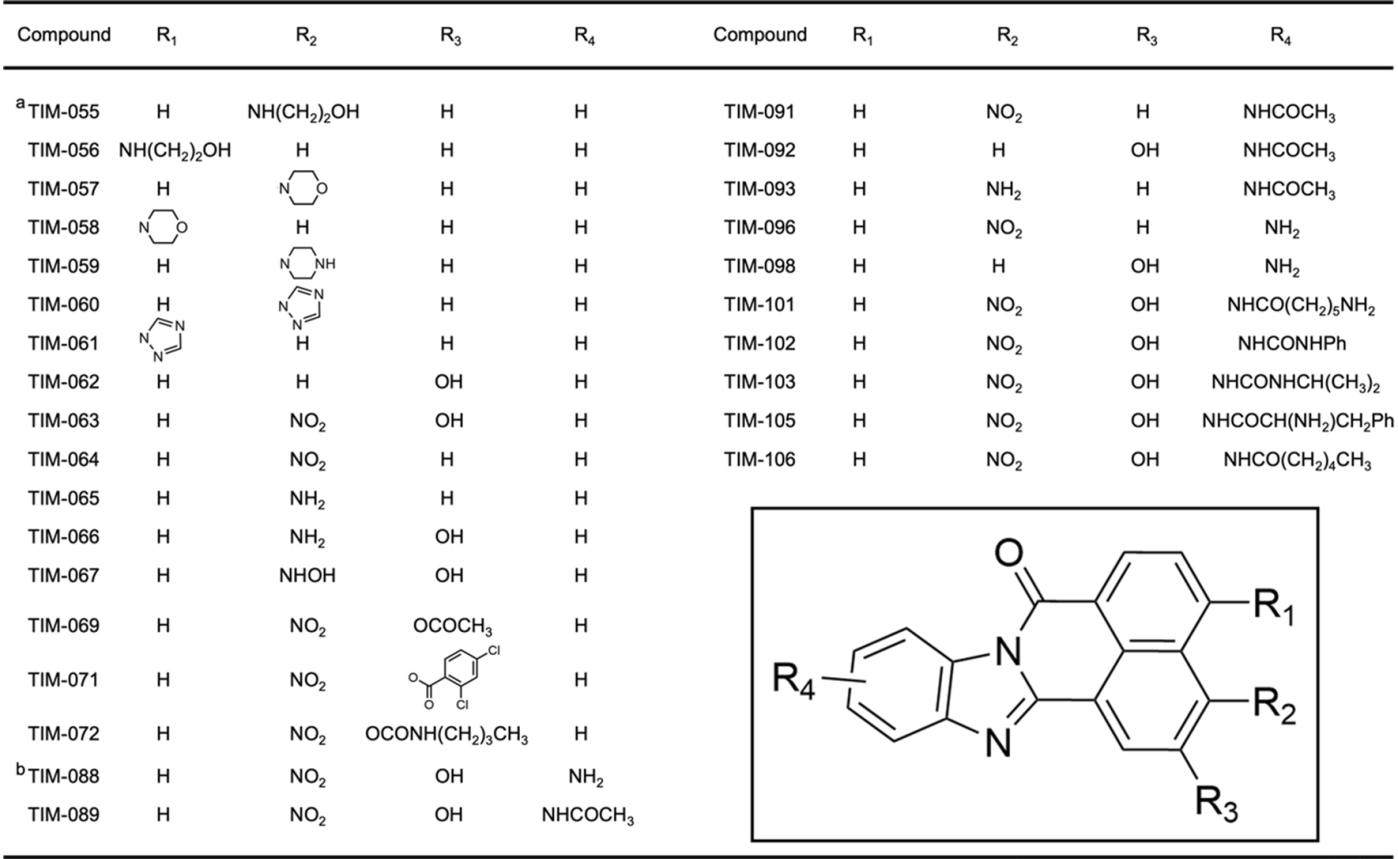
Chemical structure of TIM-063 and its derivatives with different substitutions at positions R_1_–R_4_.

^a^TIM-055–TIM-064 were synthesized as previously described^11^.

^b^TIM-088–TIM-106 were obtained as regioisomer mixtures and used without separation in kinase inhibition assays.

TIM-101 has been shown to potently inhibit AAK1 activity and inhibit the activity of CaMKK isoforms. As TIM-098 was synthesized as a mixture of four inseparable regioisomers (Table 2), we attempted to isolate the active compound. Isomer separation was difficult due to their poor solubility in organic solvents. Therefore, the mixture was converted to a more soluble tri-Boc derivative, and TIM-098a-tri-Boc was isolated as the major isomer from the mixture of other tri-Boc isomers by silica gel column chromatography. After deprotection of the tri-Boc derivatives using trifluoroacetic acid, we checked AAK1 inhibitory activities of separated TIM-098 regioisomers (see in “Methods”).

**Figure 3:**
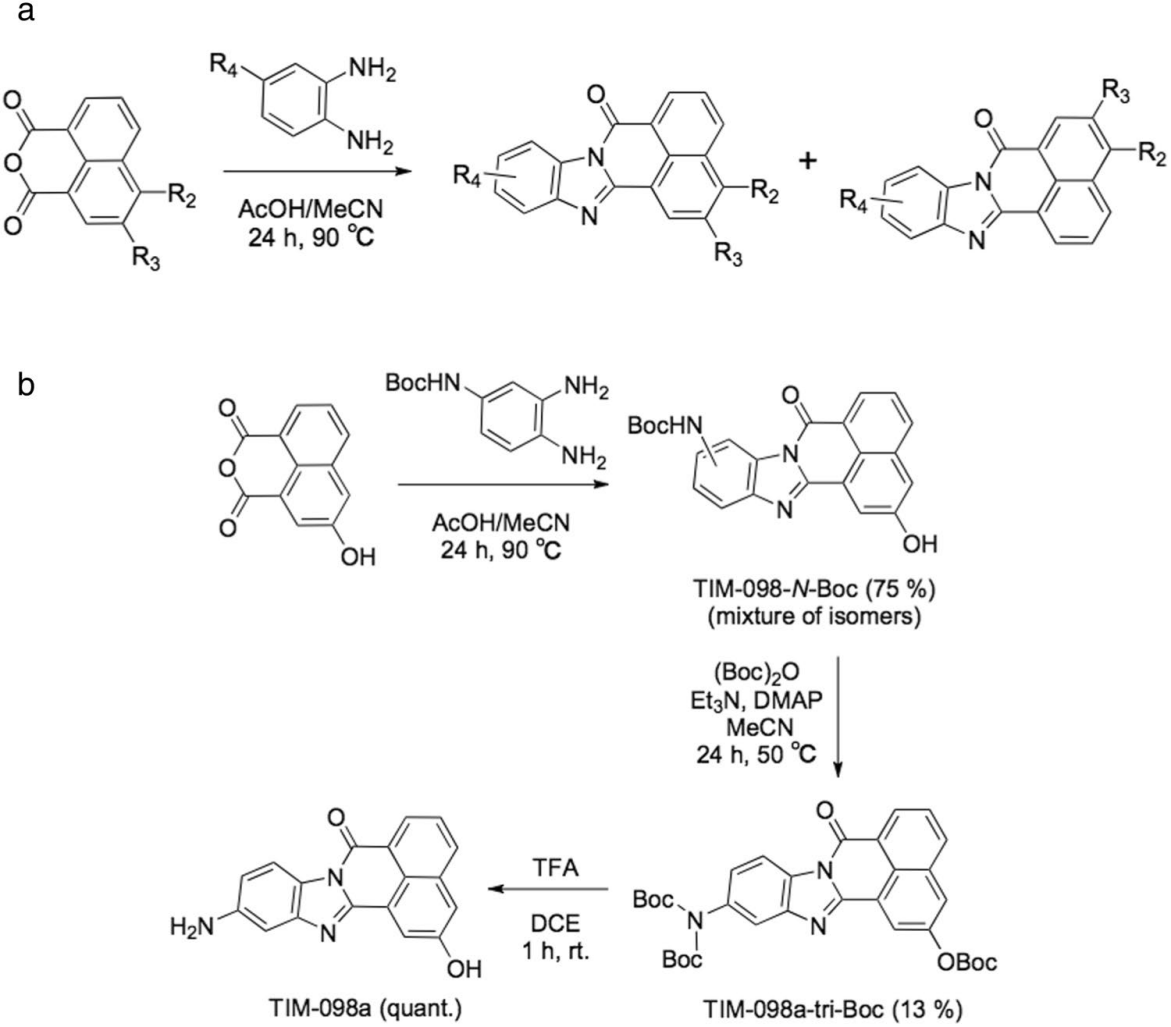
Synthesis of TIM-063 analogs (**a**) and TIM-098a (**b**).

**Figure 4:**
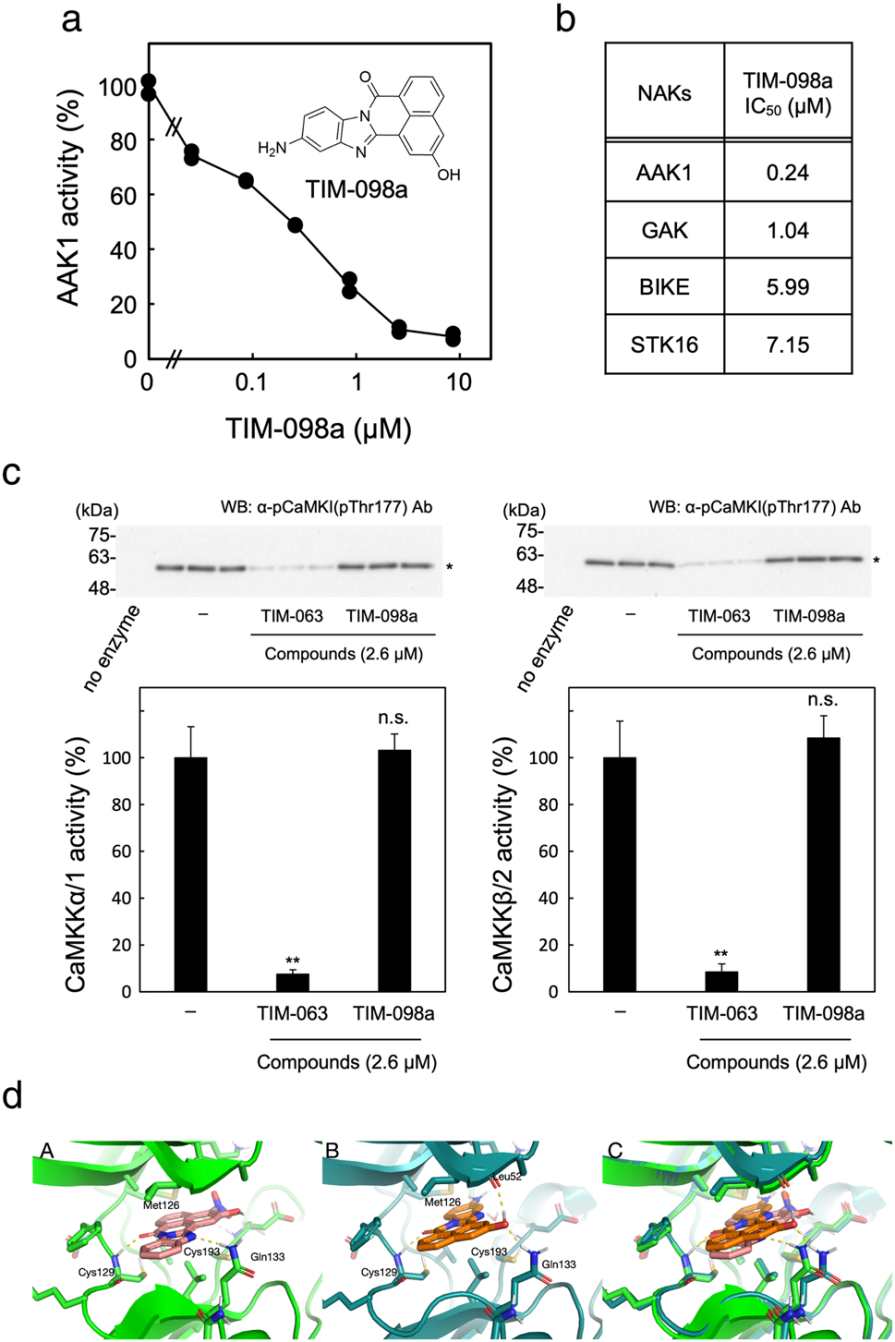
Effect of TIM-098a on AAK1, NAKs, and CaMKK activities. (**a**) Protein kinase activities of His-tagged AAK1 catalytic domain (25–396) were measured at 30 °C for 20 min using 100 μM [γ-^32^P]ATP in the presence of various concentrations of TIM-098a or absence as described in the “Methods”. AAK1 activities are quantitated and expressed as a percentage of the average value in the absence of the compound. Results represent duplicate experiments. Chemical structure of TIM-098a is indicated (*insert*). (**b**) IC_50_ value of TIM-098a against NAKs activities. Activities of NAKs, including AAK1, GAK, BIKE and STK19, were measured in the presence of various concentrations of TIM-098a in a solution described in (**a**) and the “Methods”. IC_50_ values were estimated from duplicate experiments (see Supplemental Information S3). (**c**) Activities of CaMKKα/1 (*left panels*) and β/2 (*right panels*) were measured at 30 °C for 10 min in the presence of 2.6 µM TIM-063 or TIM-098a or absence (−) via immunoblot analysis using anti-phospho-CaMKIα (pThr177) antibody (*upper panels*) as described in the “Methods”. CaMKK activities are quantitated by densitometric scanning of the immunoreactive band and expressed as a percentage of the average value in the absence of the compound (−). Results are represented as the mean ± the standard deviation indicated by error bars from triplicate experiments. Molecular mass markers (kDa) are indicated in the left lanes of immunoblot panels. Statistical differences are marked as **p < 0.05; n.s., not significant vs. CaMKK activities in the absence of compounds (−). (**d**) Energy minimized (Macromodel) Glide top scored docking poses of (A) TIM-063 and (B) TIM-098a with AAK1. (C) Overlay of (A) and (B).

Based on the AAK1 activity assay (Fig. 4a), TIM-098a (11-amino-2-hydroxy-7*H*-benzo[de]benzo[4,5]imidazo[2,1-*a*]isoquinolin-7-one; Fig. 3b, Fig. 4a inset, and Supplemental Information S2) is capable of inhibiting AAK1 with an IC_50_ value of 0.24 µM without any significant inhibitory effects against CaMKK activity up to a concentration of 2.6 µM, (Fig. 4c). It is noteworthy that the minor fraction containing other TIM-098 regioisomers did not inhibit AAK1 activity (data not shown). The inhibitory effect of TIM-098a was comparable to that of the recently developed AAK1/BMP2K inhibitor SGC-AAK1-1 (IC_50_ for AAK1 = 270 nM)^30^. AAK1 is a member of Numb-associated kinases (NAKs) that possess high sequence identity across their kinase domains^13^; thus, the inhibitory potencies of TIM-098a against other NAKs, including GAK, BIKE, and STK16, were examined (Fig. 4b).

The IC_50_ values of TIM-098a for NAKs activities indicated that the inhibitor suppressed all NAKs activities in vitro with a relative selectivity for AAK1 (4–30-fold higher inhibitory potency than that for other NAKs). Since AAK1 inhibitory activity of TIM-098a has shown to be ~ 35-fold more potent than that of a mother compound, TIM-063 (Figs. 1e and 4a), GLIDE (Schrödinger LLC) docking models of TIM-063 and TIM-098a with AAK1 were computationally simulated based on the crystal structure of a broad spectrum kinase inhibitor, K252a in complex with human AAK1 (27–365) (PDB ID: 4WSQ)^13^ (Fig. 4d and Supplemental Information S4). According to the docking models of TIM-063 (Fig. 4d, panel A) and TIM-098a (Fig. 4d, panel B) with AAK1, the two compounds likely bind with different geometries (Fig. 4d, panel C, an overlay image). We hypothesize that a plausible reason for the difference in binding pose is that the polar nitro and amino groups of TIM-063 and TIM-098a, respectively, favours placement in the same binding site region between Cys193 and Met126 (the gatekeeper residue within ATP-binding site), which requires a flipped opposite orientation of their common central tetracyclic scaffold (Fig. 4d). Although the central scaffold is flipped between the two docked complexes, it stays in the same position and maintains stacking of the π-system face to more hydrophobic top and bottom regions of the binding site. Furthermore, both docking poses reveal identical ligand carbonyl oxygen hydrogen bond with the backbone NH of Cys129 and hydrogen bonds with the side chain NH of Gln133. However, the latter hydrogen bond is involving a TIM-063 scaffold nitrogen as acceptor (Fig. 4d, panel A), while the TIM-098a involves its hydroxyl oxygen as the acceptor which at the same time allows for its hydroxyl hydrogen to donate to the backbone carbonyl of Leu52 (Fig. 4d, panel B). Hence, TIM-063 engages its carbonyl oxygen and one ring nitrogen as acceptors of two hydrogen bonds from Cys129 and Gln133, while TIM-098a in addition to accepting hydrogen bonds from Cys129 and Gln133 donates a hydrogen bond to Leu52, which may at least partly explain its enhanced affinity for AAK1.

### Effect of TIM-098a on AAK1 activity in transfected cells

**Figure 5:**
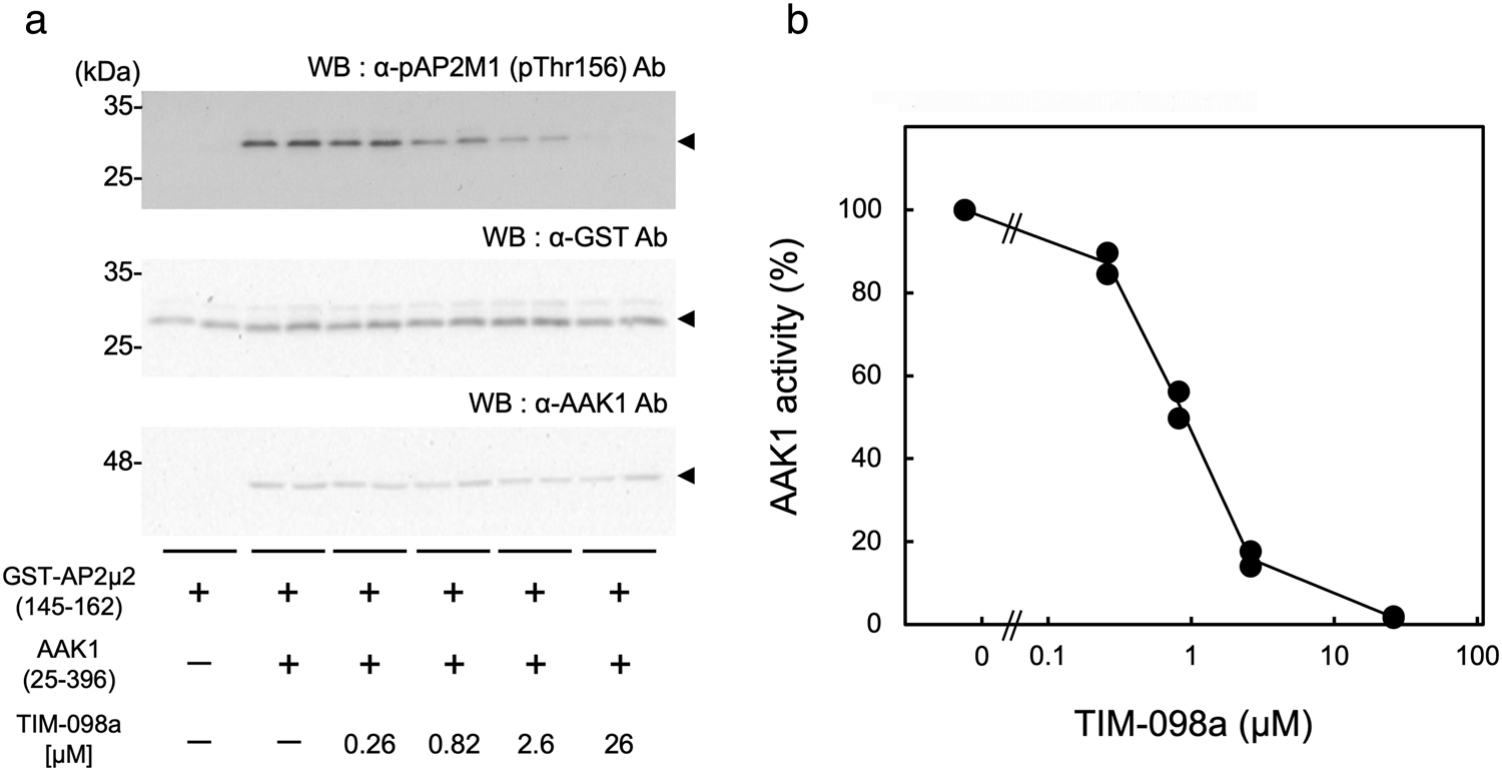
Inhibition of AAK1 activity by TIM-098a in cells. (**a**) COS-7 cells were transfected with an expression vector of GST-AP2µ2 (145–162) in the presence (+) or absence (−) of the expression vector of His-tagged AAK1 catalytic domain (25–396). After 42 h culture, the cells were cultured with or without (−) the indicated concentrations of TIM-098a for 6 h. Cell extracts were analyzed by immunoblotting using an anti-phospho-AP2µ2 (pThr156) antibody (*upper panel*), anti-GST antibody (*middle panel*), or anti-AAK1 antibody (*lower panel*). The molecular mass markers (kDa) are indicated in the left lanes of the immunoblot panels. (**b**) AAK1 activity was quantified by densitometric scanning of the immunoreactive band of pThr156 (**a**, *upper panel*) and expressed as a percentage of the average value in the absence of the compound (−). The results are representative of duplicate experiments.

To examine the effect of TIM-098a on AAK1 activity in cells, we set up an AAK1 activity assay in the cells as follows. Transfection of expression plasmids for the catalytic domain of AAK1(25–396) together with its artificial substrate (GST-AP2µ2 145–162) into COS-7 cells was followed by detection of phosphorylation of Thr156 via immunoblot analyses with anti-phospho-Thr156 antibody after 6 h-treatment with or without TIM-098a (Fig. 5). In the absence of TIM-098a, Thr156 phosphorylation of GST-AP2µ2 (145–162) was not observed without exogenous expression of AAK1, which was significantly increased by overexpression of the catalytic domain of AAK1 (25–396) (Fig. 5a). This result indicates the successful detection of AAK1 activity in the transfected cells. When we treated AAK1/GST-AP2µ2 (145–162) expressing COS-7 cells with various concentrations of TIM-098a, AAK1-catalyzed Thr156 phosphorylation of GST-AP2µ2 (145–162) was suppressed by TIM-098a treatment in a concentration-dependent manner (Fig. 5b), indicating the cell-membrane permeability and inhibitory effect of the inhibitor on AAK1 activity in the cells. Quantitative analysis indicated that TIM-098a suppressed AAK1 activity, with an IC_50_ value of 0.87 µM in transfected cells.

### Effect of TIM-098a on AAK1 overexpression-reduced early endosome formation

**Figure 6:**
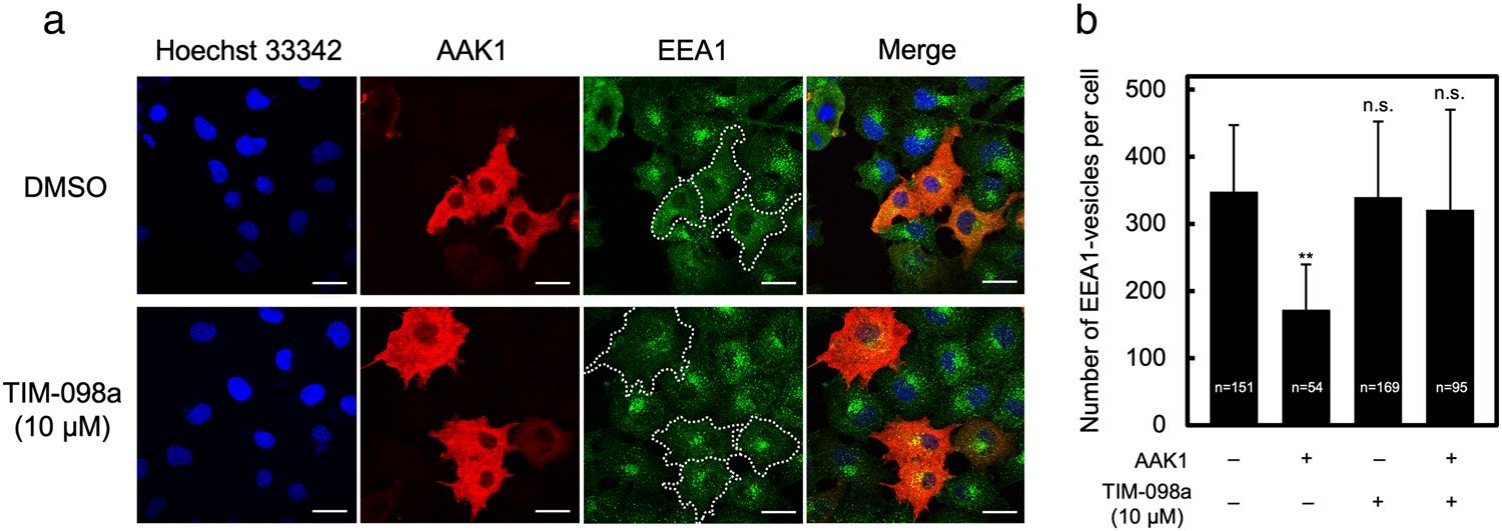
Effect of TIM-098a on AAK1 overexpression-reduced early endosome formation. HeLa cells were transfected with an expression vector containing His-tagged AAK1 (1–863). After 42 h of culture, cells were incubated with 10 µM TIM-098a-containing medium or without (DMSO) for 6 h. (**a**) Cells were fixed and immunostained with anti-AAK1 antibody (red) and anti-EEA1 antibody (green), followed by nuclear staining with Hoechst 33342 (blue). The right panels show the merged images. Scale bars = 10 µm (**b**) The number of EEA1 vesicles were quantified in 54–169 cells per condition using the ImageJ software^38^ as described in the “Methods”. The data are represented as the mean ± the standard deviation indicated by error bars. Statistical differences are marked as **p < 0.05; n.s., not significant vs. untransfected cells without TIM-098a treatment.

Because AAK1 regulates endocytosis through phosphorylation of AP2µ2^17^ and Numb^18^, we attempted to suppress the AAK1-regulated clathrin-dependent endocytosis by TIM-098a. Early endosome formation in HeLa cells was observed by immunofluorescence microscopy with an anti-early endosome antigen 1 (EEA1) antibody staining (Fig. 6a, *green*). The number of EEA1-positive early endosomes were reduced by ~ 50% in cells overexpressing full-length AAK1 observed using anti-AAK1 antibody (Fig. 6a, *red*) as compared to untransfected cells (Fig. 6a, upper panels and Fig. 6b).

This was consistent with a previous report indicating that AAK1 overexpression inhibits AP2-dependent transferrin endocytosis^32^. On treatment with 10 µM TIM-098a, the number of early endosomes in AAK1-transfected cells was recovered to the level of untransfected wild type cells (Fig. 6a, lower panels and Fig. 6b). Early endosome formation in wild type cells was not affected by this inhibitor. These results indicate that TIM-098a inhibits AAK1-regulated endocytosis by suppressing AAK1 kinase activity.

In summary, we used recently developed CaMKK inhibitor (TIM-063)–immobilized Kinobeads technology^9,10^ to search for TIM-063–interacting molecules and identified the main targets (CaMKKα/1 and β/2) and off-target kinases (AAK1 and ERK2) of TIM-063 via mass spectrometry. The inhibitory effect of TIM-063 on AAK1 was ~ tenfold lower than that on CaMKK; therefore, it seems reasonable to use TIM-063 as a CaMKK inhibitor. However, as TIM-063 is a weak inhibitor of AAK1, potent AAK1 inhibitors can be developed by screening TIM-063 derivatives. Screening chemical libraries of TIM-063 derivatives led to the discovery of TIM-098a, whose AAK1 inhibitory activity was ~ 35-fold more potent than that of TIM-063. This is in good agreement with docking analyses of both compounds with AAK1 showing that TIM-098a has the better Glide docking score than TIM-063 (see in Methods and Supplemental Information S4). TIM-098a appears to be a NAKs inhibitor that is relatively selective to AAK1 among the NAKs family and has no effect on the activity of CaMKK isoforms. TIM-098a inhibited AAK1 kinase activity in cells and AAK1-suppressed endocytosis. Taken together, these results indicated that TIM-098a, which has a chemical structure different from that of previously developed AAK1 inhibitors, may be a useful molecular probe for elucidating the physiological functions of AAK1 and evaluating the pharmacological effects of conventional AAK1 inhibitors. However, the inhibitor should be used carefully and cautiously because its specificity has not yet been completely evaluated. AAK1 has been implicated in many diseases^19–23^ and viral infections^24–26^, and inhibition of AAK1 activity is a promising therapeutic strategy; however, as a therapeutic agent, the inhibitory potency of TIM-098a against AAK1 is weaker than that of previously developed AAK1 inhibitors. Therefore, in the future, we plan to develop an AAK1 inhibitor with more selective and potent inhibitory activity using TIM-098a as a lead compound.

## Methods

### Materials

Expression plasmids for His_6_-tagged rat CaMKKα/1 (His-CaMKKα/1) and His_6_-tagged rat CaMKKβ/2 (His-CaMKKβ/2) were constructed using the pME-18s vector^33^. His-CaMKKα/1 and His-CaMKKβ/2 were expressed in COS-7 cells by transfection of both expression plasmids as described above, followed by purification with Ni-sepharose and CaM-sepharose resins. DNA fragments encoding a full-length (1–863) and the catalytic domain (25–396) of human AAK1 were amplified by reverse transcription-polymerase chain reaction (RT-PCR) using the PCR primers listed: a sense primer, 5′-atgaagaagtttttcgactcccggcgagag-3′ and antisense primer, 5′-ttaaatagccttggcttct-3′ (for AAK1 1–863) or a sense primer, 5′-ggcagcacctcgggcctgggcagtggctac-3′ (for 25–396) and antisense primer, 5′-ctaaacagtggccctcttccggggtgtcag-3′ (for AAK1 25–396) and subcloned after the N-terminal His_10_-tag in the pET-16b vector. Mammalian expression plasmid for His_10_-tagged AAK1 full-length (His-AAK1 1–863) and AAK1 catalytic domain (His-AAK1 25–396) were constructed by RT-PCR using each pET-AAK1 construct as described above as a template and PCR-primers including a sense primer, 5′-ggggaattcatgggccatcatcatcatcatcatcatcatcatcac-3′ and antisense primer, 5′-caaaaaacccctcaagacccgtttaga-3′ in the pET16 plasmid, followed by subcloning into pME-18s vector (EcoRI/XhoI). Catalytic domain cDNAs of mouse NAKs were amplified by RT-PCR using PCR-primers as follows: a sense primer, 5′-tcgctgctgcagtctgcgctgga-3′ and antisense primer, 5′-tcaccgtagggctggtcatactctgcca-3′ (for GAK 2–370); a sense primer, 5′- aagaagttctctcggatgcccaagt-3′ and antisense primer, 5′-tcaagcagggactttgacaggc-3′ (for BIKE 2–396); and a sense primer, 5′-ggccacgcactgtgtgtctgctc-3′ and antisense primer, 5′-tcagatttgggtggtgtgctggccag-3′ (for STK16 2–306) using mouse kidney cDNA as a template and subcloned into the EcoRV site of the pME-18s-His_6_ vector. His-AAK1s, His-GAK 2–370, His-BIKE 2–396, and His-STK16 2–306 were expressed in COS-7 cells by transfection with the expression plasmids described above, followed by purification using Ni-sepharose chromatography. An *Escherichia coli* expression plasmid of GST-AP2µ2 (145–162)-His_6_ was constructed by ligation of annealed DNA using a sense oligonucleotide, 5′-tcgagaaagaagagcagtcacagatcaccagccaggtaactgggcagattggctggcgg-3′ and an antisense oligonucleotide, 5′-ccgccagccaatctgcccagttacctggctggtgatctgtgactgctcttctttc-3′, into the XhoI/SmaI site of the pGEX-KG-PreS-His_6_ vector with the C-terminal His_6_-tag, followed by introduction into the BL-21 *E. coli* strain for protein expression. Recombinant GST-AP2µ2 (145–162)-His_6_ from the BL-21 *E. coli* strain was purified by Glutathione-sepharose, followed by Ni-sepharose column chromatography. The mammalian expression plasmid for GST-AP2µ2 (145–162) was constructed by PCR using pGEX-KG-PreS-AP2µ2 (145–162)-His_6_ as a template and PCR-primers as follows: a sense primer, 5′-ggggaattcatgtcccctatactaggttattgg-3′ and antisense primer, 5′-cctctagactaccgccagccaatctgcccag-3′, followed by inserting into the EcoRI/XbaI site of the pME-18s vector. Anti-phospho-CaMKIα at Thr177 (clone 9H8) was generated as previously described^34^. Anti-phospho-AP2µ2(AP2M1) at Thr156 (D4F3, 7399S) and anti-His-tag (66005-1-Ig) antibodies were obtained from Cell Signaling Technology (Danvers, MA) and Proteintech (Rosemont, IL), respectively. Anti-AAK1 (A302-145A) and anti-Erk1/2 (06-182) antibodies were obtained from Bethyl Laboratories, Inc. (Montgomery, TX) and Upstate Biotechnology, Inc. (Lake Placid, NY), respectively. Anti-EEA1 (610457) and anti-GST (27457701 V) antibodies were obtained from BD Biosciences (San Jose, CA) and GE Healthcare (Buckinghamshire, UK), respectively. Secondary antibodies conjugated to Alexa Fluorophores, Mouse IgG (H + L) Highly Cross-Adsorbed Secondary Antibody (A-11029), Rabbit IgG (H + L) Highly Cross-Adsorbed Secondary Antibody (A-11036), and Hoechst 33342 were purchased from Thermo Fisher Scientific, Inc. (Waltham, MA). BALB/c mice (aged 9–16 weeks) for the chemical proteomics analysis were purchased from Jackson Laboratory Japan, Inc. (Kanagawa, Japan). All animal procedures were approved by the Committee of Laboratory Animal Care of Okayama University (#OKU-2023520). All the experiments conducted on mice were in accordance with relevant guidelines and regulations. All methods reported in this study followed the recommendations in the ARRIVE guidelines.

### Synthesis of TIM-063 analogs

TIM-063 (2-hydroxy-3-nitro-7*H*-benzo[de]benzo[4,5]imidazo[2,1-*a*]isoquinolin-7-one) was synthesized as described previously^11^. TIM-127 (1-amino-N-(2-((2-hydroxy-3-nitro-7-oxo-7*H*-benzo[de]benzo[4,5]imidazo[2,1-*a*]isoquinolin-11-yl)amino)-2-oxoethyl)-3,6,9,12,15,18-hexaoxahenicosan-21-amide (major)) was synthesized and TIM-127-coupled sepharose was produced as described previously^12^. TIM-063 analogs were synthesized as follows. TIM-055–061 was prepared using a previously reported procedure^35^. TIM-062 and TIM-064 were prepared using a procedure similar to that used for the synthesis of TIM-063^11^. TIM-065–072 was synthesized by the reduction of the nitro group or acylation of the hydroxy group of TIM-063 or TIM-064. Other analogs, TIM-088–106, were synthesized by cyclization of the corresponding 1,8-naphthalenedicaroxylic anhydrides with 4-substituted 1,2-phenylenediamines (Fig. 3a) and further synthetic transformations, if needed, including reduction of the nitro group or deprotection of the amino group. TIM-088–106 were obtained as regioisomer mixtures and used without separation in the kinase inhibition assay. Newly synthesized compounds (TIM-065–TIM-106) were analyzed by NMR and mass spectrometry and the purity of the compounds was checked by thin-layer chromatography (Supplemental Information S2 and Fig. S2-2).

### Synthesis of TIM-098a

TIM-098a (11-amino-2-hydroxy-7*H*-benzo[de]benzo[4,5]imidazo[2,1-*a*]isoquinolin-7-one) was synthesized as follows (see Supplemental Information S2). TIM-098-*N*-*tert*-butoxycarbonyl (Boc) derivative was prepared by the cyclization reaction of 3-hydroxy-1,8-naphthalenedicarboxylic anhydride and *tert*-butyl (3,4-diaminophenyl)carbamate as a regioisomer mixture (Fig. 3b). At this stage, isomer separation was difficult because of their poor solubility in organic solvents. Therefore, the mixture was converted to a more soluble tri-Boc derivative, and TIM-098a-tri-Boc was isolated as the major isomer by silica gel column chromatography. The exact structure was determined by X-ray crystallography (Supplemental Information S2 and Fig. S2-1). TIM-098a was finally obtained by the deprotection of the tri-Boc derivative using trifluoroacetic acid.

### Cell culture and transfection

COS-7 cells and HeLa cells were obtained from American Type Culture Collection. COS-7 cells were cultured in 6-well plates or 10 cm dishes in Dulbecco’s modified Eagle’s medium supplemented with 10% fetal bovine serum at 37 °C in 5% CO_2_. COS-7 cells in 6-well plates were transfected with 0.2 µg of pME-His-AAK1 25–396 and 1.8 µg of pME-GST-AP2µ2 (145–162) expression plasmids using polyethylenimine “MAX” (Polysciences, Inc. Warrington, PA), according to the manufacturer’s protocol. After 42 h of culture, the cells were cultured in a medium with or without the indicated concentrations of TIM-098a for 6 h. The cells were then lysed with 1 × SDS-PAGE sample buffer (200 µL), followed by immunoblot analyses (10 µL cell lysates). COS-7 cells in 10 cm dishes were transfected with 10 µg of pME-His-NAKs for purification of His-tagged NAKs as described above and the binding analysis with TIM-063 Kinobeads.

### Identification of TIM-063–interacting kinases

Mouse cerebrum was extracted with 2 mL buffer A (150 mM NaCl, 20 mM Tris–HCl pH7.5, and 0.05% Tween 20) containing a protease inhibitor cocktail (1:50 dilution; Nacalai Tesque, Kyoto, Japan) followed by centrifugation to prepare cerebrum extracts. The extracts adjusted to 5 mg/mL with buffer A (450 µL) were incubated with either TIM-127-coupled sepharose or control sepharose (100 µL) in the presence of 2 mM CaCl_2_ and 6 µM CaM for 45 min at 4 °C and the resin was then washed thrice with 1 mL buffer A in the presence of 2 mM CaCl_2_. TIM-063–interacting kinases were eluted with 450 µL buffer A containing 100 µM TIM-063 and concentrated to 50 µL with Amicon Ultra-0.5 mL (Ultracel 10 K; Merck Millipore Ltd. Burlington, MA) as previously described^12^. Mass spectrometry analysis for identification of TIM-063–interacting kinases was performed as follows. Briefly, the sample eluted from TIM-127-coupled sepharose was separated by sodium dodecyl sulfate (SDS)-10% polyacrylamide gel electrophoresis (PAGE) and lightly stained with Coomassie Brilliant Blue. Next, 11 gel slices were excised from the sample lane in the range of ~17 to > 200 kDa, followed by in-gel digestion with 10 μg/mL trypsin (Promega, Madison, WI) overnight at 37 °C^36^. The digested peptides were eluted with 0.1% formic acid and were subjected to liquid chromatography-tandem mass spectrometry (LC–MS/MS) analysis, which was performed on an LCMS-IT-TOF instrument (Shimadzu, Kyoto, Japan) interfaced with a nano reverse-phase LC system (Shimadzu), as previously described^37^. MS/MS data were acquired in the data-dependent mode using LCMS solution software (Shimadzu) and converted into a single text file (containing the observed precursor peptide *m/z*, fragment ion m/z, and intensity values) using a Mascot Distiller (Matrix Science, London, UK). MS/MS data were obtained independently and merged for Mascot analysis. We set the search parameters as follows: database, SwissProt 2022_01; taxonomy, *Mus musculus*; enzyme, trypsin; Max missed cleavages, 1; fixed modifications, carbamidomethyl (Cys); variable modifications, oxidation (Met); peptide tolerance, (0.05 Da); and MS/MS tolerance, (0.05 Da).

### Binding analysis of the AAK1/TIM-063 complex

COS-7 cells transfected with expression plasmids for His-tagged AAK1 were lysed with buffer A containing protease inhibitors as described above, and then cell lysates (200 µL) were incubated with either TIM-127-coupled sepharose or control sepharose (10 µL) for 30 min. After washing the resin thrice with buffer A (500 µL), samples were eluted from the sepharose resin by incubation with 20 µL of 100 µM TIM-063 in buffer A as previously described^12^, followed by immunoblot analysis.

### NAKs activity

Purified His-AAK1 25–396 (200 ng), His-GAK 2–370 (33 ng), His-BIKE 2–396 (87 ng), and His-STK16 2–306 (95 ng) were incubated with 10 µg GST-AP2µ2 (145–162)-His_6_ in a solution (20 µL) containing 50 mM HEPES pH7.5, 10 mM Mg(CH_3_COO)_2_, 1 mM DTT, and 0.1 mM [γ-^32^P]ATP or ATP at 30 °C for the indicated time periods (His-GAK 2–370, 10 min; His-BIKE 2–396, 90 min; His-STK16 2–306, 20 min) in the presence or absence of various concentrations of inhibitors (5% DMSO). Then, 15 µL of the sample was spotted onto Whatman P81 paper and washed using 95% EtOH, followed by scintillation counting of the paper to measure the phosphate incorporation into GST-AP2µ2 (145–162)-His_6_. For immunoblot detection, reactions were terminated by the addition of an equal volume of 2 × SDS-PAGE buffer at various time points, followed by immunoblot analyses using an anti-phospho-AP2µ2 (AP2M1) at Thr156 antibody.

### CaMKK activity

Purified His-CaMKKα/1 or His-CaMKKβ/2 (40 ng) was incubated with 10 µg GST-CaMKIα 1–293KE in a solution (20 µL) containing 50 mM HEPES pH7.5, 10 mM Mg (CH_3_COO)_2_, 1 mM DTT, 2 mM CaCl_2_, 6 µM CaM, and 0.1 mM [γ-^32^P]ATP at 30 °C for a reaction time of 20 min in the presence or absence of compounds. Then, 15 µL of the sample was spotted onto Whatman P81 paper, followed by scintillation counting of the paper as described above to measure the phosphate incorporation into GST-CaMKIα 1–293KE. Reactions were terminated by the addition of an equal volume of 2 × SDS-PAGE for the immunoblot assay at 30 °C for 10 min using 2.5 ng of CaMKKs in the solution described above containing 0.1 mM ATP, followed by immunoblot analyses using anti-phospho-CaMKIα at the Thr177 antibody.

### Immunofluorescence microscopy

HeLa cells were cultured in 6-well plates containing Dulbecco’s modified Eagle’s medium supplemented with 10% fetal bovine serum at 37 °C in 5% CO_2_. The cells were transfected with 2 µg of an AAK1-expression plasmid (pME-His-AAK1 1–863) using polyethylenimine “MAX” according to the manufacturer’s protocol. After 42 h of culture, the cells were cultured with or without 10 µM TIM-098a for 6 h. HeLa cells grown on coverslips were fixed with 3.7% paraformaldehyde in PBS for 15 min and permeabilized with 0.1% Triton X-100 in PBS for 5 min at room temperature. Next, the cells were blocked with 4% bovine serum albumin (BSA) in PBS for 15 min and incubated with primary antibodies diluted in 4% BSA in PBS for 15 min. The cells were washed three times with PBS and incubated for 15 min with the appropriate secondary antibodies conjugated to Alexa fluorophores. After washing, the coverslips were mounted on microscope slides and viewed under an FV1000 confocal microscope (Olympus, Tokyo, Japan). Quantification of EEA1-positive vesicles was performed using ImageJ software^38^. EEA1 fluorescence signals with areas greater than 430 nm^2^ were counted as EEA1-positive vesicles using the particle analyzer function in ImageJ. To account for clustered EEA1 signals, the watershed segmentation method was applied in ImageJ before using the particle analyzer.

### Computational analyses

#### Ligand docking

All computational analyses were performed based on the X-ray structure of AAK1 in complex with K252a (PDB ID: 4WSQ)^13^ and with software included in the Schrödinger Maestro suite version 2023–4 (Schrödinger LLC). One protein–ligand unit was prepared with Schrödinger Protein preparation workflow-program and default settings and the Zn(II) ion complexed by Asp176 and Asp916 was kept in the complex. A binding site grid was generated with Glide with default settings and with the 4WSQ-bound K252a as the reference ligand. TIM-098a and TIM-063 were docked with Glide default settings and set to 10 poses output with top Glide scores of − 10.389 and − 9.778, respectively (Supplemental Information S4). The top scores were energy minimized in MacroModel with default settings (OPLS4 and water, Fig. 4d).

### Other analysis

Immunoblot analysis was performed using the indicated primary antibodies and horseradish peroxidase-conjugated anti-mouse IgG (NA931-1ML), anti-rabbit IgG (NA934-1ML; GE Healthcare), or anti-goat IgG (6420-05; SouthernBiotech, Birmingham, AL) as secondary antibodies. Upper and lower portions of the immunoblot membranes were cut based on molecular mass markers prior to incubation with indicated primary antibodies. A chemiluminescent reagent (PerkinElmer Life Sciences, Waltham, MA) was used for signal detection of immunoblots, followed by quantification of immunoreactivity using the ImageJ software^38^. Protein concentrations in the samples were estimated using Coomassie Brilliant Blue (Bio-Rad Laboratories, Inc.), with BSA as a standard. Student’s *t*-tests (two-tailed) were used to evaluate the statistical significance of two-group comparisons. Statistical significance was set at *p* < 0.05.

### Supplementary Information

Supplementary Information.

## Data Availability

The datasets analyzed in this study are available from the corresponding authors on reasonable request. X-ray crystallographic files (CIF) for TIM-098a tri-Boc derivative (CCDC 2306475) can be available free of charge from the Cambridge Crystallographic data Centre at https://www.ccdc.cam.ac.uk/structures/.

## Abbreviations

AP2: Adaptor protein 2
AAK1: AP2-associated protein kinase 1
CaMKK: Calcium/calmodulin-dependent protein kinase kinase
GAK: Cyclin G-associated kinase
BIKE: Bone morphogenetic protein 2-inducible kinase
STK16: Serine/threonine kinase 16
NAK: Numb-associated kinase
CaM: Calmodulin
CaMK: Calcium/calmodulin-dependent protein kinase
EEA1: Early endosome antigen 1
GST: Glutathione-*S*-transferase
RT-PCR: Reverse transcription-polymerase chain reaction
