# Supplementary material for: Development of a novel AAK1 inhibitor via Kinobeads-based screening
Source: Sci Rep. 2024 Mar 20;14:6723. doi: 10.1038/s41598-024-57051-9 (PMC10954696; doi:10.1038/s41598-024-57051-9)
Supplement: Supplementary file 1 — Supplementary Information. [file 41598_2024_57051_MOESM1_ESM.pdf]

Supplemental Information S1

Mascot Search Results for identification of TIM-063 binding protein kinases from mouse cerebrum (Table 1) as described in “Methods” are shown. Matched peptides shown in **bold red**.

(Calcium/calmodulin-dependent protein kinase kinase 2   Protein ID | KKCC2_MOUSE)

1	MSSCVSSQPT	SDR**VAPQDEL**	**GSGGGSREGQ**	**KPCEALR**GLS	SLSIHLGMES
51	FIVVTECEPG	R**GVDLNLAR**D	QPPEADGQEL	PLEASDPESR	SPLSGRKMSL
101	QEPSQGGPAS	SSNSLDMNGR	CICPSLSYSP	ASSPQSSPRM	PRRPTVESHH
151	VSITGLQDCV	QLNQYTLKDE	IGKGSYGVVK	LAYNENDNTY	YAMKVLSKKK
201	LIRQAGFPRR	PPPRGARPAP	GGCIQPRGPI	EQVYQEIAIL	KKLDHPNVVK
251	LVEVLDDPNE	DHLYMVFELV	NQGPVMEVPT	LKPLSEDQAR	**FYFQDLIK**GI
301	EYLHYQKIIH	R**DIKPSNLLV**	**GEDGHIKIAD**	**FGVSNEFK**GS	DALLSNTVGT
351	PAFMAPESLS	ETRKIFSGKA	LDVWAMGVTL	YCFVFGQCPF	MDER**IMCLHS**
401	**K**IKSQALEFP	DQPDIAEDLK	DLITR**MLDKN**	**PESR**IVVPEI	KLHPWVTRHG
451	AEPLPSEDEN	CTLVEVTEEE	VENSVK**HIPS**	**LATVILVK**TM	IRKRSFGNPF
501	EGSRREERSL	SAPGNLLTKK	PTREWEPLSE	PKEARQRRQP	PGPR**AGPCGG**
551	**GGSALVK**GGP	CVESWGAPAP	GSPPRMPPLQ	PEEVMEPE	

**Start-End**	**Observed**	**Mr(expt)**	**Mr(calc)**	**Delta**	**Miss**	**Sequence**
14-27	665.3237	1328.6328	1328.6321	0.0007	0	R.VAPQDELGSGGGSR.E
28-37	594.2886	1186.5627	1186.5764	-0.0137	0	R.EGQKPCEALR.G + Carbamidomethyl (C)
62-69	429.2492	856.4839	856.4767	0.0072	0	R.GVDLNLAR.D
291-298	537.2785	1072.5424	1072.5593	-0.0169	0	R.FYFQDLIK.G
312-327	578.9802	1733.9188	1733.9312	-0.0124	0	R.DIKPSNLLVGEDGHIK.I
328-338	613.8010	1225.5875	1225.5979	-0.0104	0	K.IADFGVSNEFK.G
395-401	444.7194	887.4243	887.4357	-0.0114	0	R.IMCLHSK.I + Carbamidomethyl (C)
426-434	545.2637	1088.5129	1088.5284	-0.0155	1	R.MLDKNPESR.I
426-434	553.2626	1104.5106	1104.5233	-0.0127	1	R.MLDKNPESR.I + Oxidation (M)
477-488	430.9338	1289.7795	1289.8071	-0.0276	0	K.HIPSLATVILVK.T
477-488	645.9065	1289.7985	1289.8071	-0.0086	0	K.HIPSLATVILVK.T
545-557	565.7775	1129.5403	1129.5550	-0.0147	0	R.AGPCGGGGSALVK.G + Carbamidomethyl (C)

(AP2-associated protein kinase 1   Protein ID | AAK1_MOUSE)

1	MKKFFDSRRE	QGSSGLGSGS	SGGGGSSSGL	GSGYIGRVFG	IGRQQVTVDE
51	VLAEGGFALV	FLVRTSNGVK	CALKRMFVNN	EHDLQVCKRE	IQIMRDLSGH
101	KNIVGYIDSS	INNVSSGDVW	EVLILMDFCR	**GGQVVNLMNQ**	**R**LQTGFTENE
151	VLQIFCDTCE	AVARLHQCKT	PIIHR**DLKVE**	**NILLHDR**GHY	VLCDFGSATN
201	KFQNPQAEGV	NAVEDEIKKY	TTLSYR**APEM**	**VNLYSGK**IIT	TKADIWALGC
251	LLYKLCYFTL	PFGESQVAIC	DGSFTIPDNS	RYSQDMHCLI	R**YMLEPDPDK**
301	RPDIYQVSYF	SFKLLKKECP	VPNVQNSPIP	AKLPEPVKAS	EAAVKKTQPK
351	ARLTDPIPTT	ETSIAPRQRP	KAGQTQPNPG	ILPIQPALTP	RKRATVQPLP
401	QAAGPSNQPG	LLPSVSQPK**A**	**QATPSQPLQS**	**SQPK**QPQAPP	TPQQTPATQT
451	QGLPTQAQAT	PQHQQQHLLK	QQQQQQQQPQ	QPTAPPQPAG	TFYQQQQQQQ
501	QQQAQTQQFQ	AVHPAAQQPV	TAQFPVGSQG	GAQQQLMQNF	YHQQQQQQQQ
551	QQQLMAQQAA	LQQKTAVVVP	QSQAQPATAP	QAAAAQEPGQ	IQAPVRQQPK
601	**VQTTPPPTIQ**	**GQK**VGSLTPP	SSPKTQRAGH	RRILSDVTHS	AVFGVPASKS
651	TQLLQAAAAE	ASLNKSK**SAT**	**TTPSGSPR**TS	QQNVSNASEG	STWNPFDDDN
701	FSK**LTAEELL**	**NKDFAK**LGEG	KLPEKLGGSA	ESLIPGFQPT	QGDAFTTPSF
751	SAGTAEKRKG	GQAVDSGIPL	LSVSDPFIPL	QVPDAPEKLI	EGLKSPDTSL
801	LLPDLLPMTD	PFGSTSDAVI	DKADVAVESL	IPGLEPPVAQ	R**LPSQTESVT**
851	**SNR**TDSLTGE	DSLLDCSLLS	NPTAGLLEEF	APIALSAPTH	KAAEDSNLIS
901	GFGVAEGSEK	VAEDEFDPIP	VLITKNTQGG	HSRNSSGSSE	SSLPNLARSL
951	LLVDQLIDL				

Start-End	Observed	Mr(expt)	Mr(calc)	Delta	Miss	Sequence
131-141	616.3047	1230.5947	1230.6139	-0.0192	0	R.GGQVVNLMNQR.L + Oxidation (M)
131-141	616.3049	1230.5953	1230.6139	-0.0186	0	R.GGQVVNLMNQR.L + Oxidation (M)
176-187	488.9389	1463.7949	1463.8096	-0.0147	1	R.DLKVENILLHDR.G
227-237	612.7966	1223.5786	1223.5856	-0.0070	0	R.APEMVNLYSGK.I + Oxidation (M)
292-300	562.2409	1122.4673	1122.4903	-0.0230	0	R.YMLEPDPDK.R + Oxidation (M)
420-434	784.3993	1566.7840	1566.8002	-0.0161	0	K.AQATPSQPLQSSQPK.Q
601-613	697.8732	1393.7319	1393.7565	-0.0247	0	K.VQTTPPPTIQGQK.V
601-613	697.8776	1393.7406	1393.7565	-0.0160	0	K.VQTTPPPTIQGQK.V
668-678	531.2568	1060.4991	1060.5149	-0.0158	0	K.SATTTPSGSPR.T
704-716	497.9335	1490.7787	1490.7980	-0.0193	1	K.LTAEELLNKDFAK.L
842-853	659.8285	1317.6425	1317.6525	-0.0099	0	R.LPSQTESVTSNR.T

(Calcium/calmodulin-dependent protein kinase kinase 1 Protein ID | KKCC1_MOUSE)

1	MESGPAVCCQ	DPRAELVDRV	AAINVAHLEE	ADEGPEPARN	GVDPPPRARA
51	ASVIPGSASR	PTPVRPSLSA	RKFSLQERPA	GSCLGAQVGP	YSTGPASHIS
101	PRSWRRPTIE	SHRVAISDTE	DCVQLNQYKL	QSEIGKGAYG	VVR**LAYNESE**
151	**DR**HYAMKVLS	KKKLLKQYGF	PRRPPPRGSQ	ATQGGPAKQL	LPLER**VYQEI**
201	**AILK**KLDHVN	VVKLIEVLDD	PAEDNLYLVF	DLLRKGPVME	VPCDKPFPEE
251	QARLYLRDII	LGLEYLHCQK	IVHRDIKPSN	LLLGDDGHVK	IADFGVSNQF
301	EGNDAQLSST	AGTPAFMAPE	AISDSGQSFS	GKALDVWATG	VTLYCFVYGK
351	CPFIDDYILT	LHRKIKNEAV	VFPEEPEVSE	DLKDLILR**ML**	**DKNPETR**IGV
401	SDIKLHPWVT	KHGEEPLPSE	EEHCSVVEVT	EEEVKNSVRL	IPSWTTVILV
451	KSMLRKRSFG	NPFEPQARRE	ERSMSAPGSL	LMKEGCGEGC	KSPELPGVQE
501	DEAAS				

Start-End	Observed	Mr(expt)	Mr(calc)	Delta	Miss	Sequence
144-152	548.7464	1095.4782	1095.4832	-0.0050	0	R.LAYNESEDR.H
196-204	538.8161	1075.6177	1075.6277	-0.0100	0	R.VYQEIAILK.K
389-397	560.2740	1118.5335	1118.5390	-0.0055	1	R.MLDKNPETR.I + Oxidation (M)

(Mitogen-activated protein kinase 01 Protein ID | MK01_MOUSE)

1	MAAAAAAGPE	MVRGQVFDVG	PRYTNLSYIG	EGAYGMVCSA	YDNLNKVRVA
51	IKKISPFEHQ	TYCQRTLREI	KILLRFRHEN	IIGINDIIRA	PTIEQMKDVY
101	IVQDLMETDL	YKLLKTQHLS	NDHICYFLYQ	ILRGLKYIHS	ANVLHRDLKP
151	SNLLLNTTCD	LKICDFGLAR	VADPDHDHTG	FLTEYVATRW	YR**APEIMLNS**
201	**K**GYTKSIDIW	SVGCILAEML	SNRPIFPGKH	YLDQLNHILG	ILGSPSQEDL
251	NCIINLKAR**N**	**YLLSLPHK**NK	VPWNRLFPNA	DSKALDLLDK	MLTFNPHKRI
301	EVEQALAHPY	LEQYYDPSDE	PIAEAPFKFD	MELDDLPKEK	LKELIFEETA
351	RFQPGYRS				

Start-End	Observed	Mr(expt)	Mr(calc)	Delta	Miss	Sequence
193-201	509.7608	1017.5071	1017.5164	-0.0093	0	R.APEIMLNSK.G + Oxidation (M)
260-268	542.8079	1083.6012	1083.6077	-0.0064	0	R.NYLLSLPHK.N

Supplemental Information S2

The NMR spectra were recorded on a Varian 400MR with the solvent peak ($CDCl_3$: δ H 7.26; DMSO-d_6: δ H 2.50; acetonitrile-d_3: δ H 1.93) as the internal reference. The multiplicities were indicated as s (singlet), d (doublet), t (triplet), q (quartet), m (multiplet), and br (broad), and the coupling constants (*J*) were expressed in Hertz (Hz). The X-ray crystallographic analysis was performed on RIGAKU ValiMax Saturn equipped with HyPix detector. The mass spectra were recorded on an Agilent Q-TOF G6520. Thin-layer chromatography analysis was performed on a Merck TLC Silica gel 60 F254.

Synthesis of TIM-098 *N-tert*-butoxycarbonyl derivative

tert-Butyl (3,4-diaminophenyl)carbamate (217 mg, 1.01 mmol), 3-hydroxy-1,8-naphthalenedicarboxylic anhydride (222 mg, 0.99 mmol) and acetic acid (1 mL, 17.2 mmol) were dissolved in acetonitrile (3 mL). The mixture was heated at 90 °C for 24 hours in a sealed tube. The obtained precipitate was filtered and washed with mixed solvent of ethyl acetate-hexane to yield TIM-098-*N*-Boc derivative (301 mg, 0.75 mmol, 75 %, brown solid), as a mixture of regioisomers.

Synthesis of TIM-098a tri-(*tert*-butoxycarbonyl) derivative

TIM-098-*N*-Boc derivative (301 mg, 0.75 mmol), di-*tert*-butyl dicarbonate (518 mg, 2.25 mmol), triethylamine (415 µl, 3.00 mmol) and 4-dimethylaminopyridine (10 mg, 0.075 mmol) were dissolved in acetonitrile (4 mL). The mixture was stirred at 60 °C for 24 hours. The solvent was removed by evaporation and the obtained residue was purified by column chromatography on silica gel using mixed solvent of ethyl acetate-hexane to give TIM-098a tri-Boc derivative (61 mg, 0.10 mmol, 13 %, yellow solid) and mixture of other isomers (131 mg, 28 %). Recrystallization of TIM-098a tri-Boc was performed using mixed solvent of diethyl ether-hexane.
^{1}H NMR (400 MHz, $CDCl_3$) δ 1.40 (s, 18H), 1.62 (s, 9H), 7.28 (dd, 1H, *J* = 2.1, 8.4 Hz), 7.66 (d, 1H, *J* = 2.0 Hz), 7.86 (dd, 1H, *J* = 7.6, 8.4 Hz), 7.96 (d, 1H, *J* = 2.0 Hz), 8.28 (d, 1H, *J* = 2.1, 7.6 Hz), 8.53 (d, 1H, *J* = 8.6 Hz), 8.69 (d, 1H, *J* = 2.0 Hz), 8.79 (dd, 1H, *J* = 2.0, 8.6 Hz).

Synthesis of TIM-098a (11-amino-2-hydroxy-7*H*-benzo[de]benzo[4,5]imidazo[2,1-*a*]isoquinolin-7-one)

A solution of TIM-098a tri-Boc derivative (10.0 mg, 0.017 mmol) in 1,2-dichloroethane (1 mL) was added trifluoroacetic acid (0.40 mL, 5.2 mmol). The mixture was stirred for 1 hour at room temperature and concentrated *in vacuo* to yield TIM-098a (5.1 mg, quantitative, red oil).
^{1}H NMR (400 MHz, DMSO-d_6) δ 6.94 (d, 1H, *J* = 8.6 Hz), 7.20 (brs, 1H), 7.58 (d, 1H, *J* = 2.1 Hz), 7.79 (dd, 1H, *J* = 7.6, 8.6 Hz), 8.19 (d, 1H, *J* = 7.6 Hz), 8.20 (d, 1H, *J* = 2.1 Hz), 8.29 (d, 1H, *J* = 8.6 Hz), 8.44 (d, 1H, *J* = 7.6 Hz), 10.55 (s, 1H); ESI-MS, *m/z*: 302 $(M+1)^+$.

Other isomers of TIM-098 were prepared from the mixture of other TIM-098 tri-Boc isomers by the similar procedure as that for TIM-098a.

X-ray crystallographic analysis

Single crystals of TIM-098a tri-Boc derivative $C_{33}H_{36}N_3O_8$ were prepared by recrystallization from diethyl ether. A suitable crystal was selected and mounted on a RIGAKU ValiMax with Saturn equipped with a HyPix detector using a MicroLoop™. The crystal was kept at 110 K during data collection. The data were processed using the CrysAllis software package[1]. Using Olex2[2], the structure was solved with the olex2.solve[3] structure solution program using Charge Flipping and refined on F^2 (with all independent reflections) with the SHELXL[4] refinement package using Least Squares minimization.

Initially in the analysis of this compound, an asymmetric unit contains one molecule and some water oxygens as a crystal solvent. However, the positions of water molecules could not be determined because they are disordered in close proximity to each other. Therefore, the electron densities of water molecules are removed using SQUEEZE[5] treatment of PLATON[6]. The resulting solvent accessible void volume per unit cell is 758 Å^3 (11.4% of unit cell volume) with the electron counts of 146 which is roughly correspond to 14.5 water molecules (145 electrons).

The ORTEP drawing of this compound is shown in Figure S2-1 and crystallographic parameters, bond distances, and bond angles are collected in Table S2-1, S2-2, and S2-3, respectively.

X-ray crystallographic files (CIF) for TIM-098a tri-Boc derivative (CCDC 2306475) can be available free of charge from the Cambridge Crystallographic data Centre at https://www.ccdc.cam.ac.uk/structures/.

1. CrysAlisPro 1.171.42.74a (Rigaku Oxford Diffraction, 2022).
2. Dolomanov, O.V., Bourhis, L.J., Gildea, R.J, Howard, J.A.K. & Puschmann, H. (2009), J. Appl. Cryst. 42, 339−341.
3. Bourhis, L.J., Dolomanov, O.V., Gildea, R.J., Howard, J.A.K., Puschmann, H. (2015). Acta Cryst. A71, 59−75.
4. Sheldrick, G.M. (2015). Acta Cryst. C71, 3−8.
5. A. L. Spek, *Acta Crystallogr. Sect. C−Struct. Chem.,* **2015**, *71*, 9.
6. A. L. Spek, *J. Appl. Cryst.*, **2003**, *36*, 7.

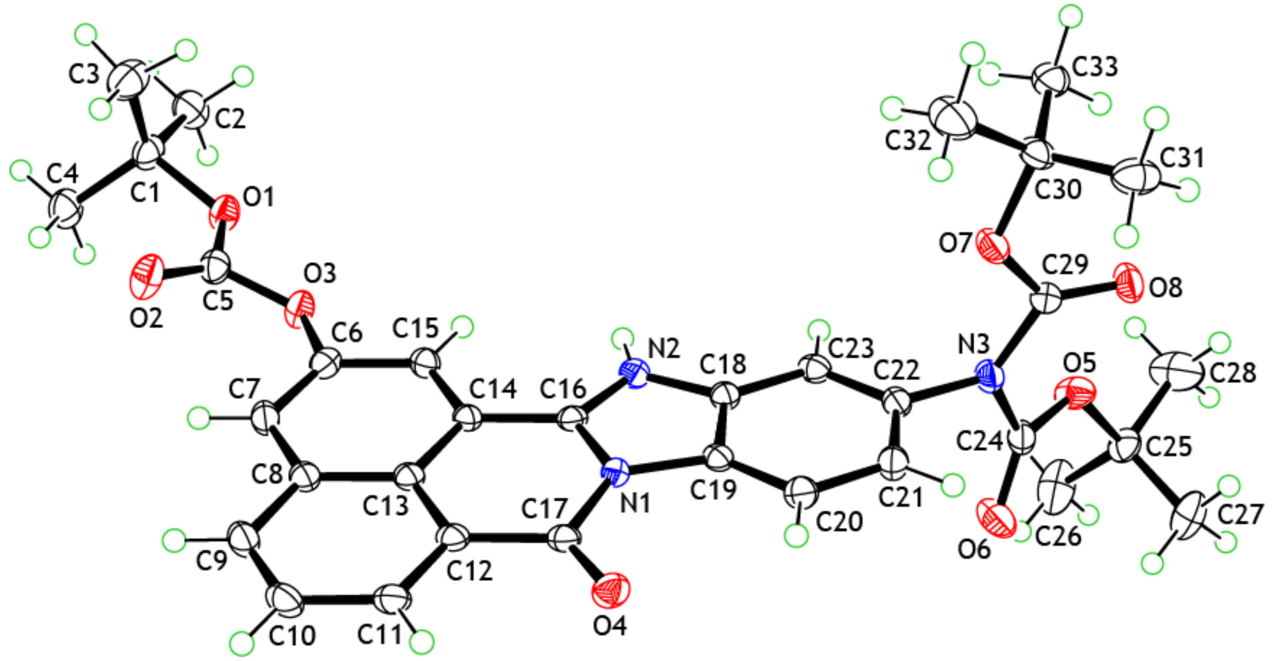

Figure S2-1. The ORTEP drawing of TIM-098a tri-Boc derivative with 50% probability ellipsoids.

Table S2-1: Crystal data and structure refinement for TIM-098a tri-Boc derivative.

Empirical formula	$C_{33}H_{36}N_3O_8$
Formula weight	602.65
Temperature/K	110
Crystal system	orthorhombic
Space group	*Aea*2
a/Å	38.2811(15)
b/Å	29.1270(8)
c/Å	5.9398(2)
a/°	90
b/°	90
g/°	90
Volume/Å^3	6623.0(4)
Z	8
*r*calc g/cm^3	1.209
μ/mm^{-1}	0.087
F(000)	2552.0
Crystal size/mm^3	0.33 × 0.082 × 0.055
Radiation	Mo Ka (λ = 0.71073)
2θ range for data collection/°	5.594 to 59.18
Index ranges	$-49 \le h \le 40$, $-40 \le k \le 29$, $-8 \le l \le 6$
Reflections collected	18899
Independent reflections	6896 [R_{int} = 0.0270, R_{sigma} = 0.0312]
Data/restraints/parameters	6896/1/406
Goodness of fit on F^2	1.039
Final *R* indexes [*I*>=2s (*I*)]	R_1 = 0.0320, wR_2 = 0.0789
Final *R* indexes [all data]	R_1 = 0.0357, wR_2 = 0.0809
Largest diff. peak/hole / e Å^{-3}	0.21/−0.38

Table S2-2: Bond Lengths for TIM-098a tri-Boc derivative.

Atom−Atom	Length/Å	Atom−Atom	Length/Å
O4−C17	1.216(2)	C14−C13	1.424(2)
O3−C5	1.357(2)	C14−C15	1.371(2)
O3−C6	1.396(2)	C23−C22	1.381(3)
O7−C29	1.332(2)	C17−C12	1.481(2)
O7−C30	1.495(2)	C13−C12	1.418(3)
O1−C5	1.324(2)	C13−C8	1.422(2)
O1−C1	1.489(2)	C12−C11	1.376(2)
O2−C5	1.195(2)	C15−C6	1.406(2)
O8−C29	1.201(2)	C20−C21	1.398(2)
O6−C24	1.201(2)	C8−C9	1.420(3)
O5−C25	1.484(2)	C8−C7	1.420(3)
O5−C24	1.322(2)	C11−C10	1.408(3)
N1−C16	1.399(2)	C22−C21	1.399(3)
N1−C19	1.408(2)	C9−C10	1.371(3)
N1−C17	1.407(2)	C6−C7	1.366(3)
N2−C18	1.393(2)	C25−C27	1.502(3)
N2−C16	1.302(2)	C25−C28	1.514(3)
N3−C22	1.452(2)	C25−C26	1.511(3)
N3−C29	1.409(2)	C1−C3	1.516(3)
N3−C24	1.411(2)	C1−C4	1.520(3)
C18−C19	1.407(2)	C1−C2	1.524(3)
C18−C23	1.395(2)	C30−C33	1.516(3)
C16−C14	1.450(2)	C30−C32	1.519(3)
C19−C20	1.381(2)	C30−C31	1.522(3)

Table S2-3: Bond Angles for TIM-098a tri-Boc derivative.

Atom−Atom−Atom	Angle/˚	Atom−Atom−Atom	Angle/˚
C5−O3−C6	122.56(14)	C12−C11−C10	120.43(18)
C29−O7−C30	119.37(13)	C23−C22−N3	118.14(16)
C5−O1−C1	120.21(14)	C23−C22−C21	122.21(16)
C24−O5−C25	120.11(13)	C21−C22−N3	119.63(16)
C16−N1−C19	105.98(14)	O1−C5−O3	104.75(15)
C16−N1−C17	125.37(14)	O2−C5−O3	126.16(18)
C17−N1−C19	128.36(15)	O2−C5−O1	129.08(17)
C16−N2−C18	105.05(15)	C10−C9−C8	121.12(17)
C29−N3−C22	121.58(14)	O3−C6−C15	111.61(15)
C29−N3−C24	122.52(14)	C7−C6−O3	126.57(16)
C24−N3−C22	115.71(13)	C7−C6−C15	121.69(17)
N2−C18−C19	111.22(14)	C6−C7−C8	119.44(16)
N2−C18−C23	128.70(17)	C9−C10−C11	120.41(17)
C23−C18−C19	120.06(16)	O7−C29−N3	108.54(14)
N1−C16−C14	118.92(15)	O8−C29−O7	127.10(16)
N2−C16−N1	113.47(15)	O8−C29−N3	124.33(16)
N2−C16−C14	127.59(16)	C20−C21−C22	121.04(17)
C18−C19−N1	104.29(15)	O5−C25−C27	109.25(16)
C20−C19−N1	132.83(16)	O5−C25−C28	101.93(15)
C20−C19−C18	122.88(15)	O5−C25−C26	110.13(16)
C13−C14−C16	118.31(16)	C27−C25−C28	111.72(19)
C15−C14−C16	121.26(15)	C27−C25−C26	112.81(18)
C15−C14−C13	120.43(16)	C26−C25−C28	110.5(2)
C22−C23−C18	117.37(17)	O6−C24−O5	126.59(18)
O4−C17−N1	120.12(15)	O6−C24−N3	121.46(17)
O4−C17−C12	125.09(16)	O5−C24−N3	111.89(14)
N1−C17−C12	114.78(15)	O1−C1−C3	109.12(16)
C12−C13−C14	121.25(15)	O1−C1−C4	109.50(15)
C12−C13−C8	120.05(15)	O1−C1−C2	101.41(15)
C8−C13−C14	118.70(16)	C3−C1−C4	113.48(17)
C13−C12−C17	121.05(15)	C3−C1−C2	110.98(17)
C11−C12−C17	118.96(17)	C4−C1−C2	111.64(17)
C11−C12−C13	119.99(16)	O7−C30−C33	108.44(15)
C14−C15−C6	120.02(16)	O7−C30−C32	101.77(14)

C19−C20−C21	116.42(16)	O7−C30−C31	110.30(15)
C9−C8−C13	118.00(17)	C33−C30−C32	110.52(17)
C7−C8−C13	119.70(16)	C33−C30−C31	113.57(15)
C7−C8−C9	122.30(16)	C32−C30−C31	111.56(19)

NMR and mass spectral data of new compounds (TIM-065–106) were listed bellow;

TIM-065

^{1}H NMR (400 MHz, acetonitrile-d_3) δ 5.95–6.15 (br, 2H), 6.84 (dt, 1H, *J* = 1.2, 7.6 Hz), 6.94 (dd, 1H, *J* = 1.2, 8.2 Hz), 6.96 (d, 1H, *J* = 8.2 Hz), 7.08 (dd, 1H, *J* = 1.2, 8.2 Hz), 7.22 (dt, 1H, *J* = 1.2, 7.6 Hz), 7.71 (dd, 1H, *J* = 7.3, 8.5 Hz), 8.31 (d, 1H, *J* = 8.2 Hz), 8.41 (dd, 1H, *J* = 1.2, 8.5 Hz), 8.53 (dd, 1H, *J* = 1.2, 7.3 Hz); ESI-MS, *m/z*: 286 $(M+H)^+$.

TIM-066

^{1}H NMR (400 MHz, acetonitrile-d_3) δ 6.92 (dt, 1H, *J* = 1.2, 7.9 Hz), 7.00 (dd, 1H, *J* = 1.2, 8.2 Hz), 7.12 (dd, 1H, *J* = 1.2, 7.9 Hz), 7.28 (dt, 1H, *J* = 1.5, 8.2 Hz), 7.68 (dd, 1H, *J* = 7.3, 8.5 Hz), 8.06 (s, 1H), 8.38 (dd, 1H, *J* = 0.9, 8.5 Hz), 8.42 (dd, 1H, *J* = 0.9, 7.3 Hz), 10.10–10.90 (br, 3H); ESI-MS, *m/z*: 302 $(M+H)^+$.

TIM-067

^{1}H NMR (400 MHz, acetonitrile-d_3) δ 5.97–6.20 (br, 2H), 6.80 (dt, 1H, *J* = 1.2, 7.6 Hz), 6.89 (dd, 1H, *J* = 1.2, 8.2 Hz), 7.10 (dd, 1H, *J* = 1.2, 8.2 Hz), 7.23 (dt, 1H, *J* = 1.2, 7.6 Hz), 8.50 (dd, 1H, *J* = 7.3, 8.4 Hz), 8.49 (d, 1H, *J* = 8.3 Hz), 8.66 (d, 1H, *J* = 8.3 Hz), 8.72 (dd, 1H, *J* = 1.2, 8.4 Hz), 8.82 (dd, 1H, *J* = 1.2, 7.3 Hz); ESI-MS, *m/z*: 302 $(M+H)^+$.

TIM-069

^{1}H NMR (400 MHz, $CDCl_3$) δ 2.43 (s, 3H), 6.92–7.00 (m, 2H), 7.19 (dd, 1H, *J* = 1.2, 7.9 Hz), 7.36 (dd, 1H, *J* = 1.2, 8.2 Hz), 7.98 (dd, 1H, *J* = 7.3, 8.5 Hz), 8.25 (d, 1H, *J* = 8.5 Hz), 8.51 (s, 1H), 8.77 (d, 1H, *J* = 7.3 Hz); ESI-MS, *m/z*: 374 $(M+H)^+$

TIM-071

^{1}H NMR (400 MHz, $CDCl_3$) δ 7.21–7.30 (m, 2H), 7.34 (dd, 1H, *J* = 1.2, 7.9 Hz), 7.43 (dd, 1H, *J* = 1.2, 8.2 Hz), 7.44 (dd, 1H, *J* = 2.1, 8.5Hz), 7.61 (d,1H, *J* = 2.1 Hz), 8.03 (dd, 1H, *J* = 7.3, 8.6 Hz), 7.61 (d,1H, *J* = 8.5 Hz), 8.32 (dd, 1H, *J* = 0.9, 8.6 Hz), 8.66 (s, 1H), 8.78 (dd, 1H, *J* = 0.9, 7.3 Hz); ESI-MS, *m/z*: 504 $(M+H)^+$.

TIM-072

^{1}H NMR (400 MHz, $CDCl_3$) δ 1.24 (t, 3H, *J* = 7.5 Hz), 1.40–1.52 (m, 4H), 3.02–3.18 (m, 2H), 7.22–7.42 (m,

3H), 7.50 (dd, 1H, *J* = 1.2, 7.9 Hz), 8.01 (dd, 1H, *J* = 7.3, 8.5 Hz), 8.46 (s, 1H), 8.62 (dd, 1H, *J* = 0.9, 7.3 Hz), 9.18 (dd, 1H, *J* = 0.9, 8.5 Hz), 11.32–11.41 (br, 1H); ESI-MS, *m/z*: 431 $(M+H)^+$.

TIM-088

^{1}H NMR (400 MHz, DMSO-d_6) for major isomer: δ 5.20–5.24 (br, 2H), 7.16 (dd, 1H, *J* = 2.0, 8.6 Hz), 7.32 (d, 1H, J = 2.0 Hz), 7.95 (dd, 1H, *J* = 7.2, 8.5 Hz), 8.16 (dd, 1H, *J* = 1.0, 8.5 Hz), 8.21 (d, 1H, *J* =8.6 Hz), 8.42 (s, 1H), 8.52 (dd, 1H, *J* = 1.0, 7.2 Hz), 12.01–12.36 (br, 3H); ESI-MS, *m/z*: 347 $(M+H)^+$.

TIM-089

^{1}H NMR (400 MHz, DMSO-d_6) for major isomer: δ 2.09 (s, 3H), 7.54 (dd, 1H, *J* = 2.0, 8.8 Hz), 7.95 (dd, 1H, *J* = 7.2, 8.5 Hz), 8.06 (dd, 1H, *J* = 0.9, 8.5 Hz), 8.27 (d, 1H, *J* = 2.0 Hz), 8.28 (d, 1H, *J* = 8.8 Hz), 8.42 (s, 1H), 8.51 (dd, 1H, *J* = 0.9, 7.2 Hz), 10.35 (brs, 1H), 12.10–12.40 (br, 1H); ESI-MS, *m/z*: 389 $(M+H)^+$.

TIM-091

^{1}H NMR (400 MHz, DMSO-d_6) for major isomer: δ 2.09 (s, 3H), 7.54 (dd, 1H, *J* = 2.0, 8.8 Hz), 8.10 (dd, 1H, *J* = 7.2, 8.5 Hz), 8.18 (d, 1H, *J* = 8.7 Hz), 8.55 (dd, 1H, *J* = 0.9, 8.5 Hz), 8.57 (d, 1H, *J* = 8.7 Hz), 8.72–8.62 (m, 3H), 10.16 (br, 1H); ESI-MS, *m/z*: 373 $(M+H)^+$.

TIM-092

^{1}H NMR (400 MHz, DMSO-d_6) for major isomer: δ 2.09 (s, 3H), 7.55 (dd, 1H, *J*= 2.0, 8.7 Hz), 7.58 (d, 1H, *J*= 2.1 Hz), 8.14 (dd, 1H, *J*= 7.6, 8.6 Hz), 8.19 (d, 1H, *J* = 8.7 Hz), 8.20 (brs, 1H), 8.22 (d, 1H, *J* = 2.1 Hz), 8.28 (d, 1H, *J*= 1.2, 8.6 Hz), 8.45 (dd, 1H, *J*= 1.2, 7.6 Hz), 10.15 (brs, 1H), 10.50–10.68 (br, 1H); ESI-MS, *m/z*: 344 $(M+H)^+$.

TIM-093

^{1}H NMR (400 MHz, DMSO-d_6) for major isomer: δ 2.09 (s, 3H), 5.90–6.25 (br, 2H), 6.92 (d, 1H, *J* = 8.3 Hz), 7.55 (dd, 1H, *J* = 1.9, 8.7 Hz), 7.76 (dd, 1H, *J* = 7.2, 8.6 Hz), 8.27 (d, 1H, *J* = 8.7 Hz), 8.31 (d, 1H, *J* = 8.3 Hz), 8.52 (dd, 1H, *J* = 1.2, 8.5 Hz), 8.63–8.78 (m, 2H); ESI-MS, *m/z*: 343 $(M+H)^+$.

TIM-096

^{1}H NMR (400 MHz, DMSO-d_6) for major isomer: δ 7.13 (dd, 1H, *J* = 1.9, 8.7 Hz), 7.39 (d, 1H, *J* = 1.9 Hz), 8.15 (dd, 1H, *J* = 7.6, 8.6 Hz), 8.25 (d, 1H, *J* = 8.7 Hz), 8.56 (dd, 1H, *J* = 2.1, 8.6 Hz), 8.60 (d, 1H, *J*= 8.7 Hz), 8.76-8.85 (m, 2H), 9.30-9.70 (br, 2H); ESI-MS, *m/z*: 331 $(M+H)^+$.

TIM-101

^{1}H NMR (400 MHz, DMSO-d_6) for major isomer: δ 1.30–1.70 (m, 6H), 2.30–2.50 (m, 2H), 2.78–2.82 (m, 2H), 7.50–7.60 (m, 3H), 7.98 (dd, 1H, *J* = 7.3, 8.5 Hz), 8.03 (dd, 1H, *J* = 1.0, 8.5 Hz), 8.06 (d, 1H, *J* = 1.8 Hz),

8.21 (d, 1H, *J* = 8.6 Hz), 8.41 (s, 1H), 8.52 (dd, 1H, *J* = 1.0, 7.3 Hz), 10.09 (brs, 1H), 12.25–12.57 (br, 1H); ESI-MS, *m/z*: 460 $(M+H)^+$.

TIM-102

^1H NMR (400 MHz, DMSO-d_6) for major isomer: δ 7.02–8.10 (m, 11H), 8.22 (dd, 1H, *J* = 1.0, 8.5 Hz), 8.43 (s, 1H), 8.52 (dd, 1H, *J* = 1.0, 7.3 Hz), 11.85–12.16 (br, 1H); ESI-MS, *m/z*: 466 $(M+H)^+$.

TIM-103

^1H NMR (400 MHz, DMSO-d_6) for major isomer: δ 1.15 (d, 6H, *J* = 7.6 Hz), 3.77–4.20 (m, 1H), 6.02–6.10 (br, 2H), 7.38 (dd, 1H, *J* = 2.0, 8.6 Hz), 7.98 (dd, 1H, *J* = 7.3, 8.5 Hz), 8.07 (d, 1H, *J* = 8.6 Hz), 8.20 (d, 1H, *J* = 2.0 Hz), 8.22 (dd, 1H, *J* = 1.0, 8.5 Hz), 8.41 (s, 1H), 8.50 (dd, 1H, *J* = 1.0, 7.3 Hz), 11.80–12.10 (br, 1H); ESI-MS, *m/z*: 432 $(M+H)^+$.

TIM-105

^1H NMR (400 MHz, DMSO-d_6) for major isomer: δ 3.02–3.40 (m, 2H), 4.15–4.22 (m, 1H), 7.20–7.40 (m, 3H), 7.49 (dd, 1H, *J* = 2.0, 8.6 Hz), 7.90 (dd, 1H, *J* = 7.2, 8.5 Hz), 8.10 (dd, 1H, *J* = 1.0, 8.5 Hz), 8.20–8.50 (m, 4H), 8.42 (s, 1H), 8.56 (dd, 1H, *J* = 1.0, 7.2 Hz), 10.60 (brs, 1H), 12.25–12.60 (br, 1H); ESI-MS, *m/z*: 494 $(M+H)^+$.

TIM-106

^1H NMR (400 MHz, DMSO-d_6) for major isomer: δ 0.93 (t, 3H, *J* = 7.5 Hz), 1.24–1.40 (m, 4H), 1.58–1.67 (m, 2H), 2.30–2.40 (m, 2H), 7.57 (dd, 1H, *J* = 2.0, 8.6 Hz), 7.96 (dd, 1H, *J* = 7.2, 8.5 Hz), 8.24 (dd, 1H, *J* = 1.0, 8.5 Hz), 8.24 (d, 1H, *J* = 8.6 Hz), 8.40 (d, 1H, *J* = 2.0 Hz), 8.44 (s, 1H), 8.53 (dd, 1H, *J* = 1.0, 7.2 Hz), 10.12 (brs, 1H), 12.05–12.45 (br, 1H); ESI-MS, *m/z*: 445 $(M+H)^+$.

Thin-layer chromatography (TLC) analysis was performed to check the purity of newly synthesized compounds (TIM-065–TIM-106) used in kinase inhibition assays (developing solvent: ethyl acetate, detection: 365 nm UV irradiation).

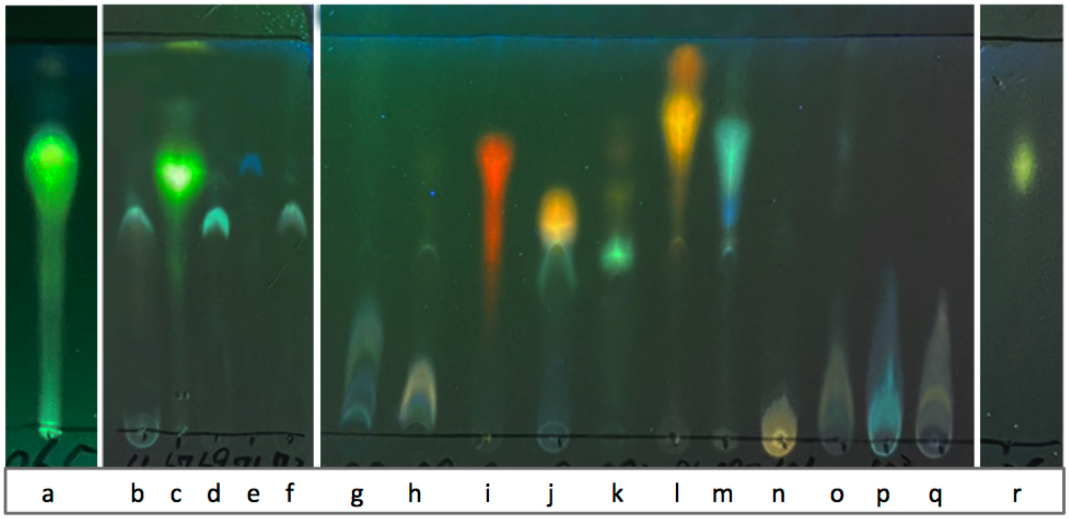

Figure S2-2. TLC analysis of newly synthesized compounds.

a, TIM-065; b, TIM-066; c, TIM-067; d, TIM-069; e, TIM-071; f, TIM-072; g, TIM-088; h, TIM-089; i, TIM-091; j, TIM-092; k, TIM-093; l, TIM-096; m, TIM-098; n, TIM-101; o, TIM-102; p, TIM-103; q, TIM-105; r, TIM-106

Supplemental Information S3

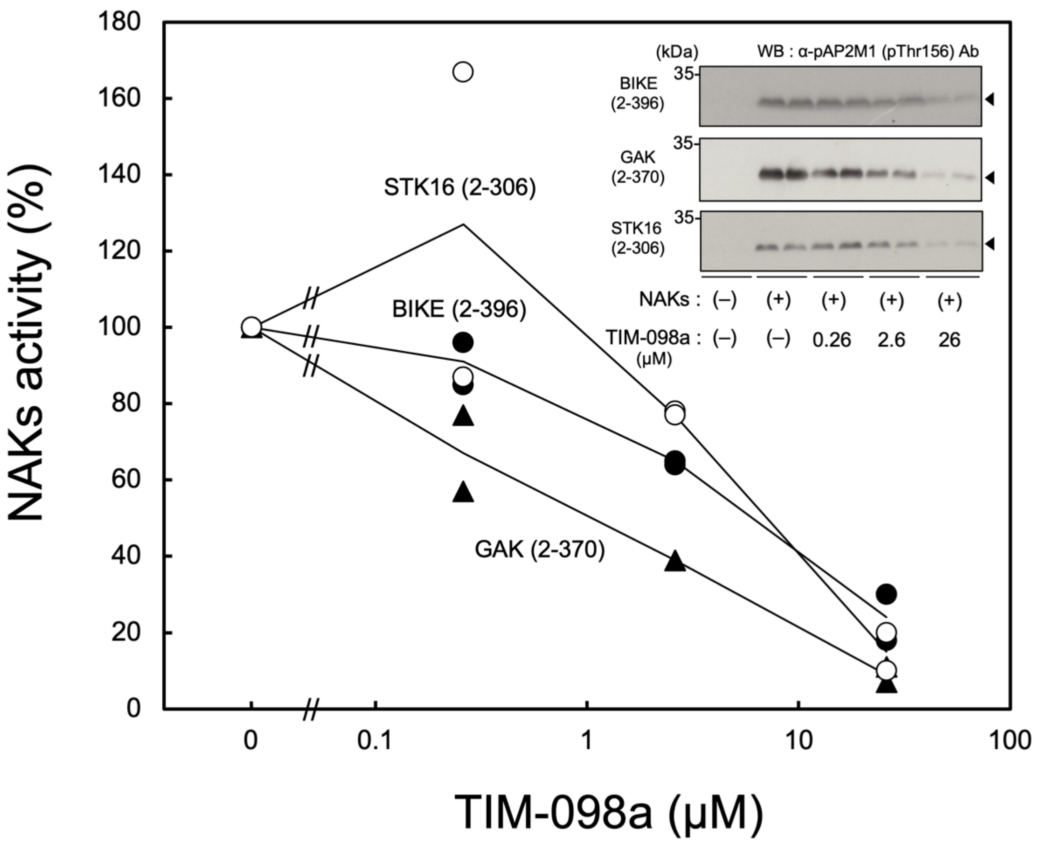

Effect of TIM-98a on NAKs' activities. Purified His-GAK 2–370 (33 ng, closed triangles), His-BIKE 2–396 (87 ng, closed circles), and His-STK16 2–306 (95 ng, open circles) were incubated with 10 μg GST-AP2μ2 (145–162)-His_6 in a solution (20 μL) containing 50 mM HEPES pH7.5, 10 mM $Mg(CH_3COO)_2$, 1 mM DTT, and 0.1 mM ATP at 30°C for the indicated time periods (His-GAK 2–370, 10 min; His-Bike 2–396, 90 min; His-STK16 2–306, 20 min) in the presence or absence (-) of various concentrations of TIM-098a. Then the reactions were terminated by the addition of an equal volume of 2× SDS-PAGE buffer, followed by immunoblot analyses using anti-pThr156 of the AP2M1 antibody (insert panels). NAK's activities are quantitated by densitometric scanning of the immunoreactive bands and expressed as a percentage of the average value in the absence of the compound (-). Results represent duplicate experiments. Molecular mass markers (kDa) are indicated in the left lanes of immunoblot panels.

Supplemental Information S4

Glide Output Data

Title	PDB ID	glide rotatable bonds	docking score	glide ligand efficiency	glide ligand efficiency sa
TIM-098a top score	4WSQ	1	-10.389	-0.452	-1.285
TIM-063 top score	4WSQ	2	-9.778	-0.391	-1.144
TIM-063 6th score	4WSQ	2	-9.531	-0.381	-1.115

Title	glide ligand efficiency ln	glide gscore	glide lipo	glide hbond	glide rewards
TIM-098a top score	-2.512	-10.389	-4.777	-0.602	-2.093
TIM-063 top score	-2.318	-9.778	-4.214	-0.217	-2.656
TIM-063 6th score	-2.259	-9.531	-4.959	0.000	-2.228

Title	glide evdw	glide ecoul	glide erotb	glide esite	glide emodel
TIM-098a top score	-35.135	-7.739	0.000	0.000	-68.187
TIM-063 top score	-44.690	-3.043	0.000	0.000	-76.005
TIM-063 6th score	-44.517	-0.784	0.000	0.000	-72.430

Title	glide energy	glide einternal	glide posenum
TIM-098a top score	-42.874	3.194	336
TIM-063 top score	-47.732	0.288	233
TIM-063 6th score	-45.301	1.141	368

(TIM-063 6th score has the same binding mode as TIM-098a top score)

Supplemental Information S5: uncropped westernblot panels

Figure 1 b, upper and middle panels

WB: anti-CaMKKβ Ab

(Upper panel)

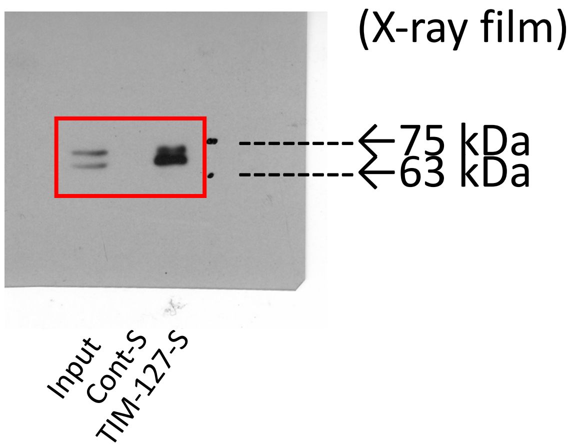

WB: anti-AAk1 Ab

(Middle panel)

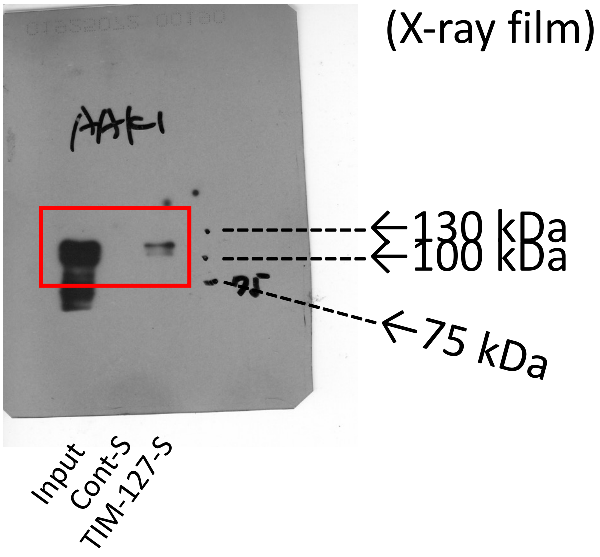

The western blot membranes were cut prior to incubation with indicated primary antibodies to detect the inhibitor-interacting kinases in the samples.

Figure 1 b, bottom panel

WB: anti-Erk1/2 Ab

(Bottom panel)

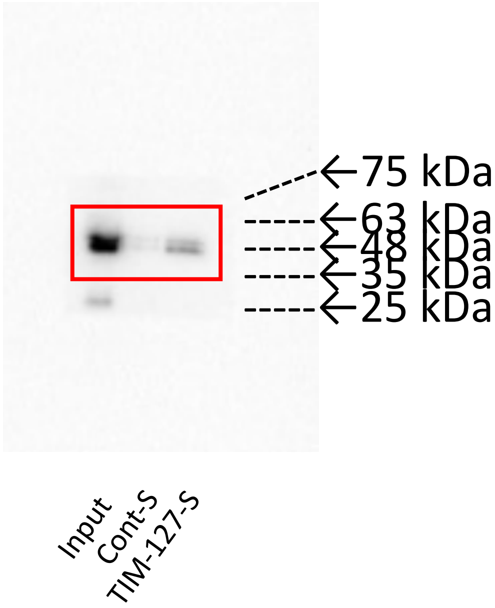

The western blot membrane was cut prior to incubation with indicated anti-Erk1/2 antibody to detect the inhibitor-interacting kinase in the samples.

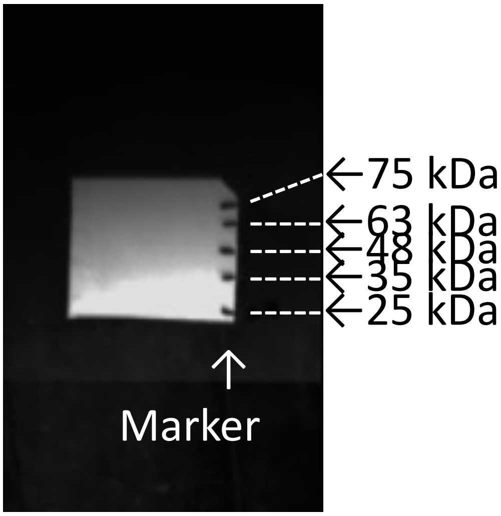

Blotted membranes with pre-stained molecular mass markers were visualized by ChemiDoc XRS (Bio-Rad Laboratories, Inc., Hercules, CA).

Figure 1 c

WB: anti-AAK1 Ab

The western blot membrane was cut prior to incubation with anti-AAK1 antibody to detect AAK1in the samples.

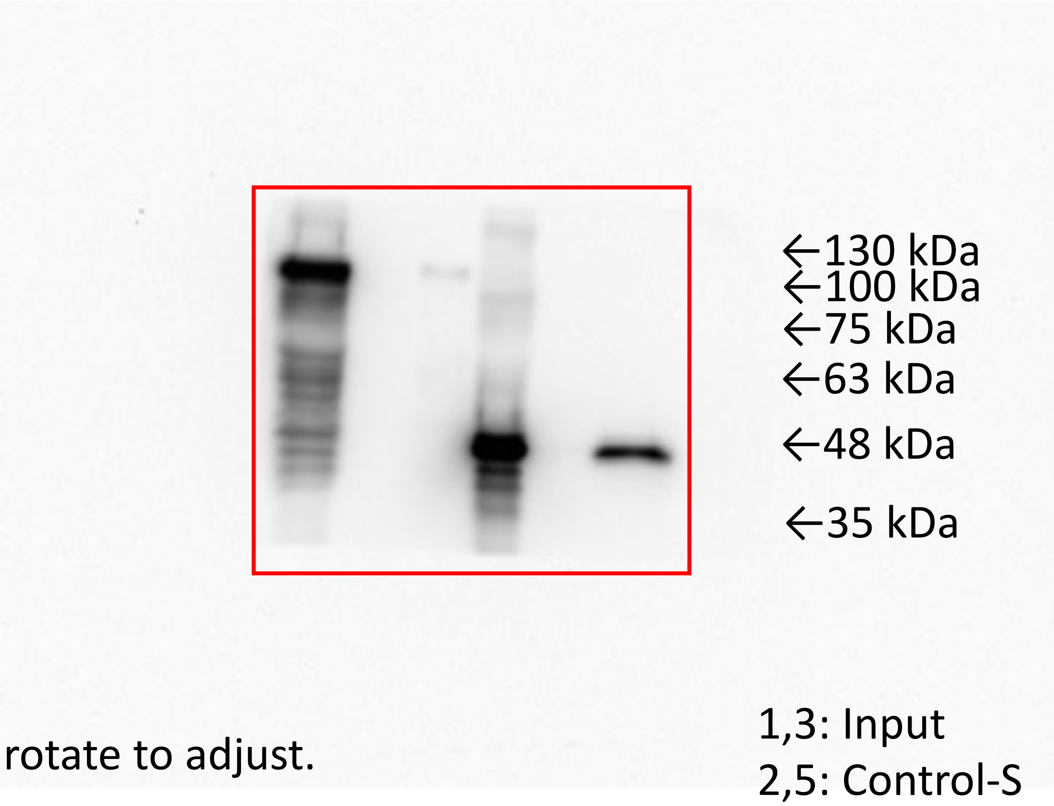

*Slightly rotate to adjust.

1 2 3 4 5 6

3,6: TIM-127-S

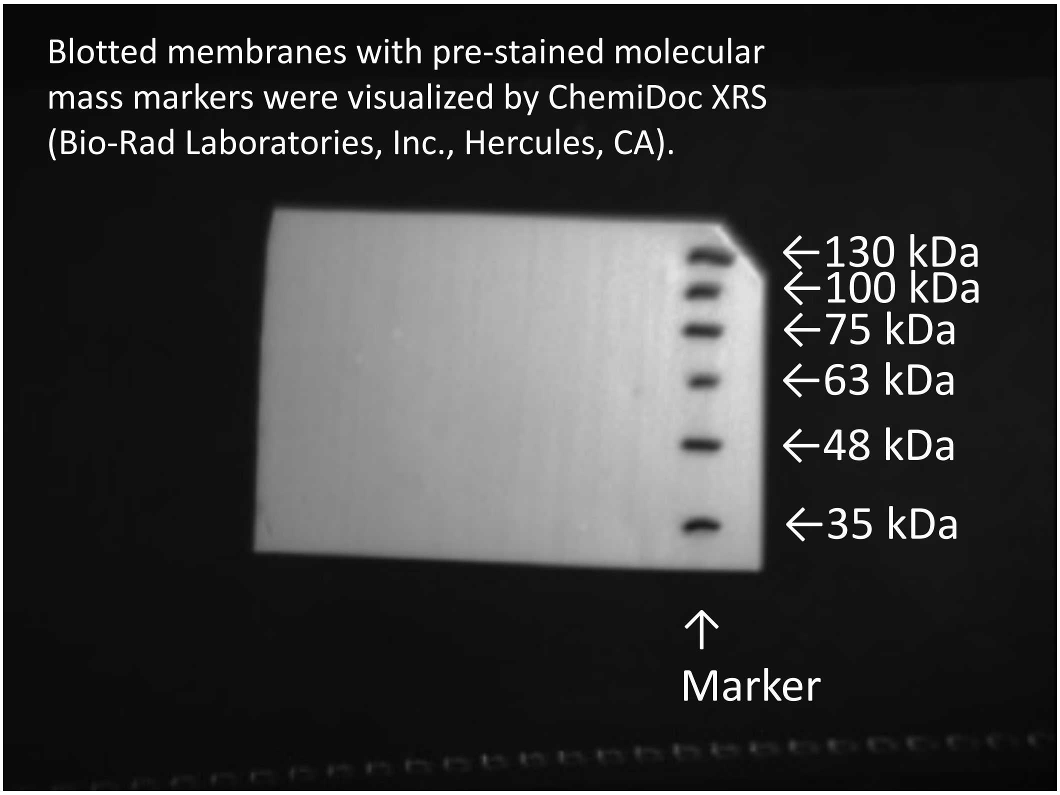

Figure 1 d, insert

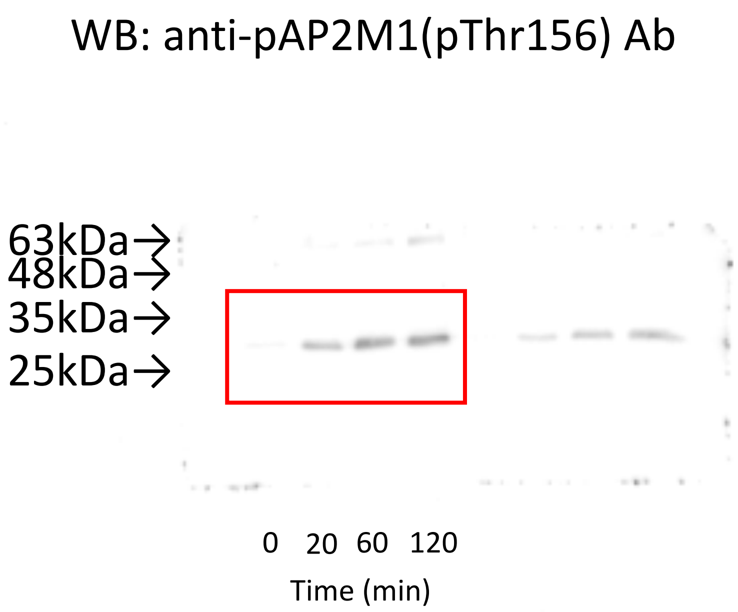

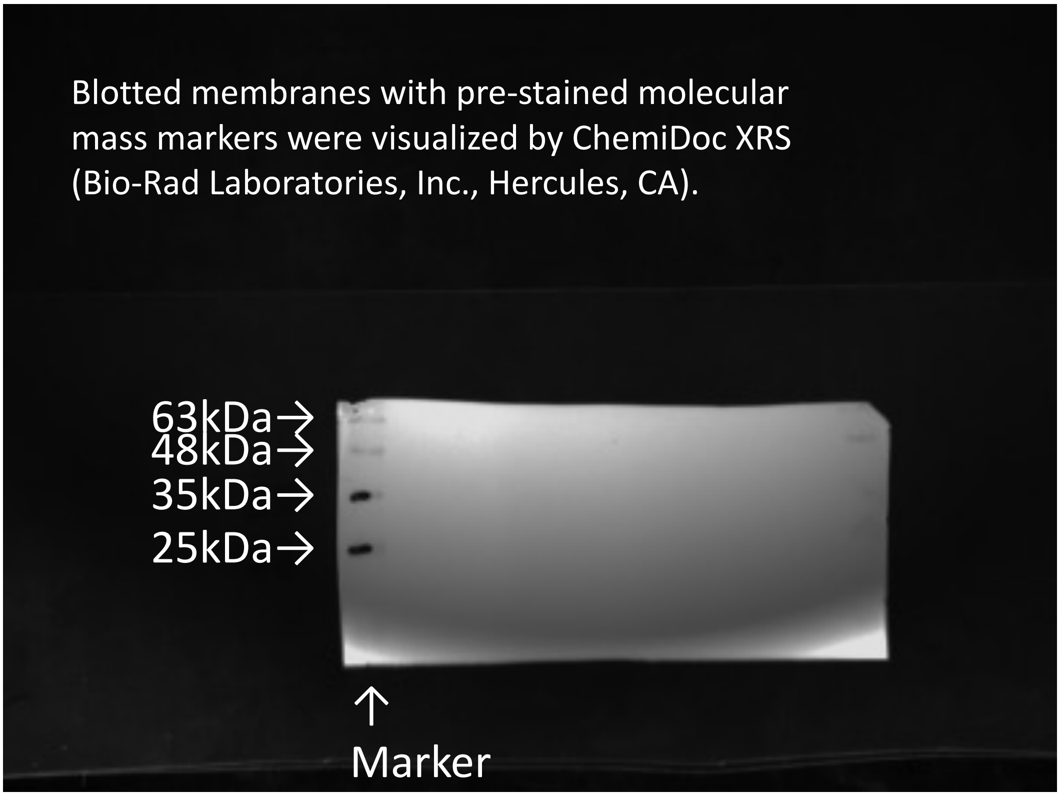

The western blot membrane was cut prior to incubation with anti-pAP2µ2M1 antibody to examine the phosphorylation level of the substrate protein (GST-AP2µ2 145-162) by AAK1 (25-396).

Figure 4 c, upper panels

WB: anti-pCaMKI (pThr177) Ab

The western blot membranes were cut prior to incubation with anti-pCaMKI antibody to examine the phosphorylation level of the substrate protein (GST-CaMKI1-293KE) by CaMKK.

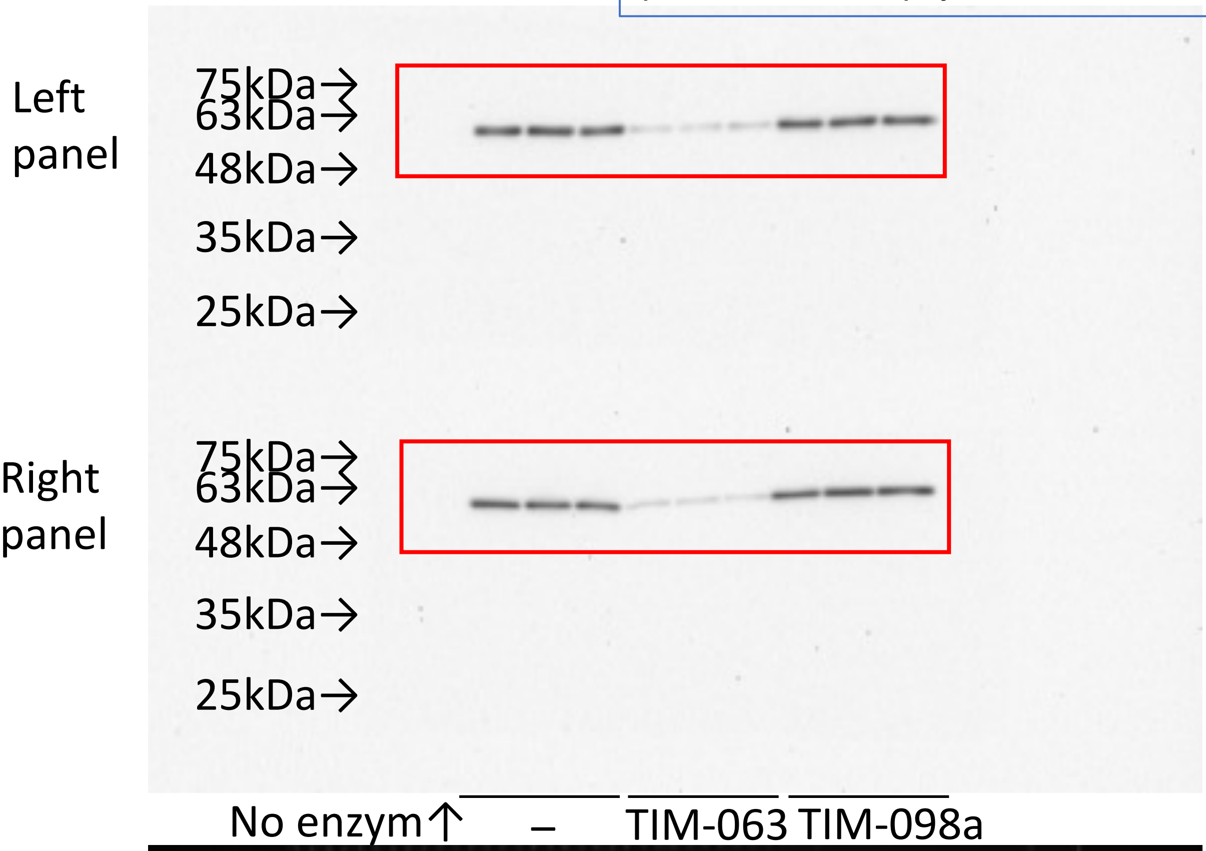

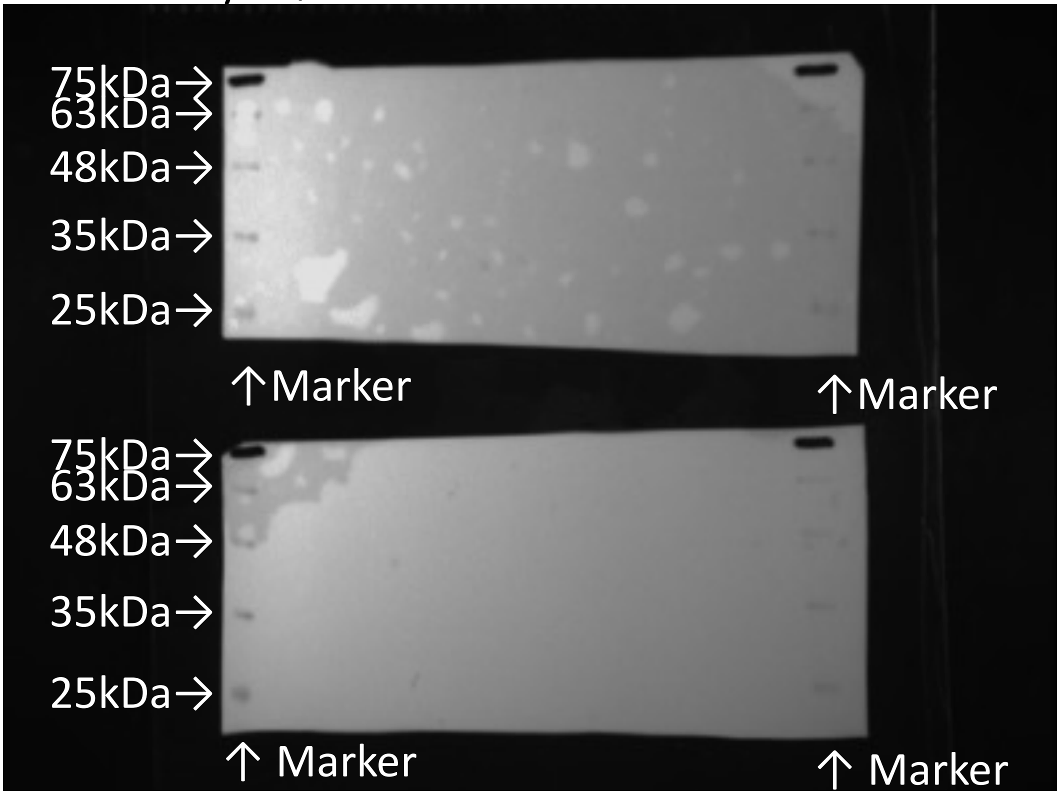

Blotted membranes with pre-stained molecular mass markers were visualized by ChemiDoc XRS (Bio-Rad Laboratories, Inc., Hercules, CA).

Figure 5 a, upper panel

WB: anti-pAP2M1(pThr156) Ab

(X-ray film)

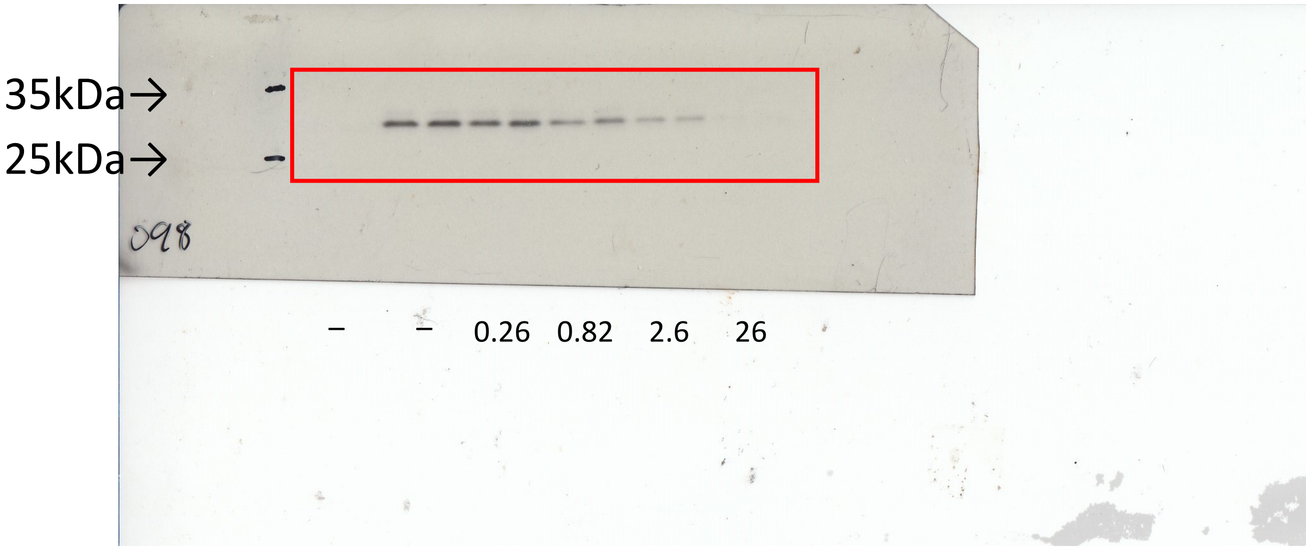

The western blot membrane was cut prior to incubation with anti-pAP2μ2M1 antibody to examine the phosphorylation level of the substrate protein (GST-AP2μ2 145-162) in cell lysates.

Figure 5a, middle panel

WB: anti-GST Ab

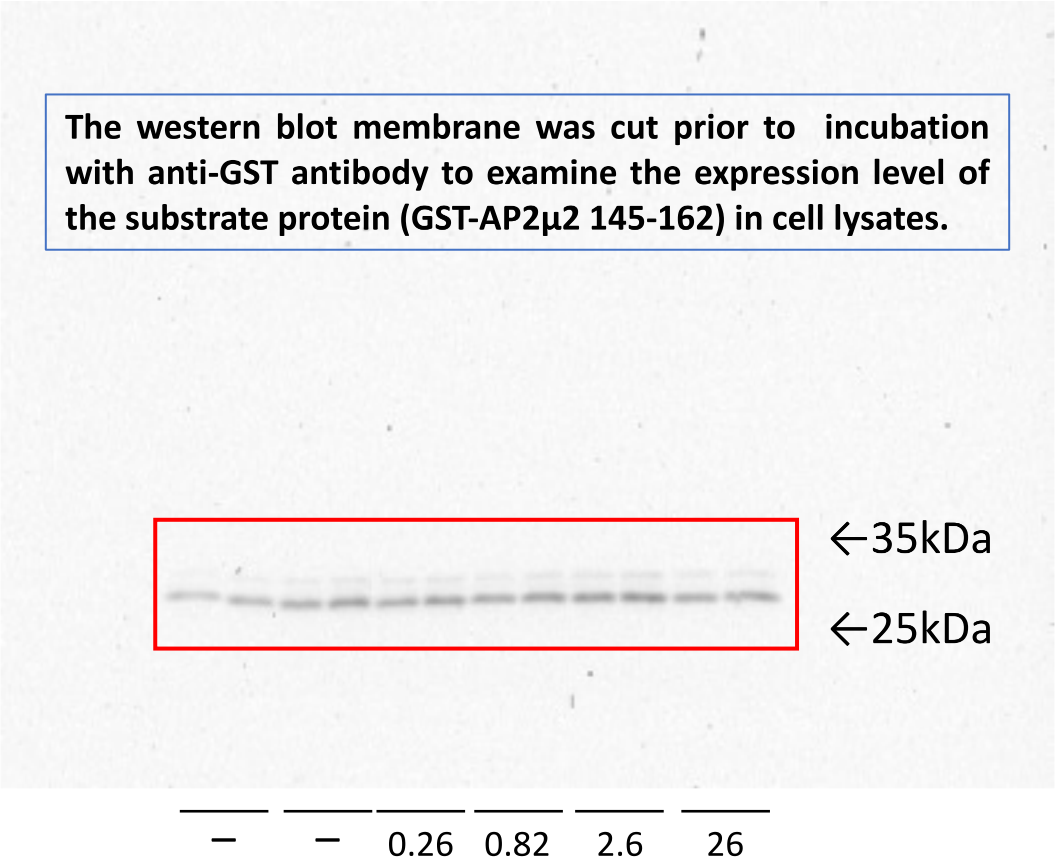

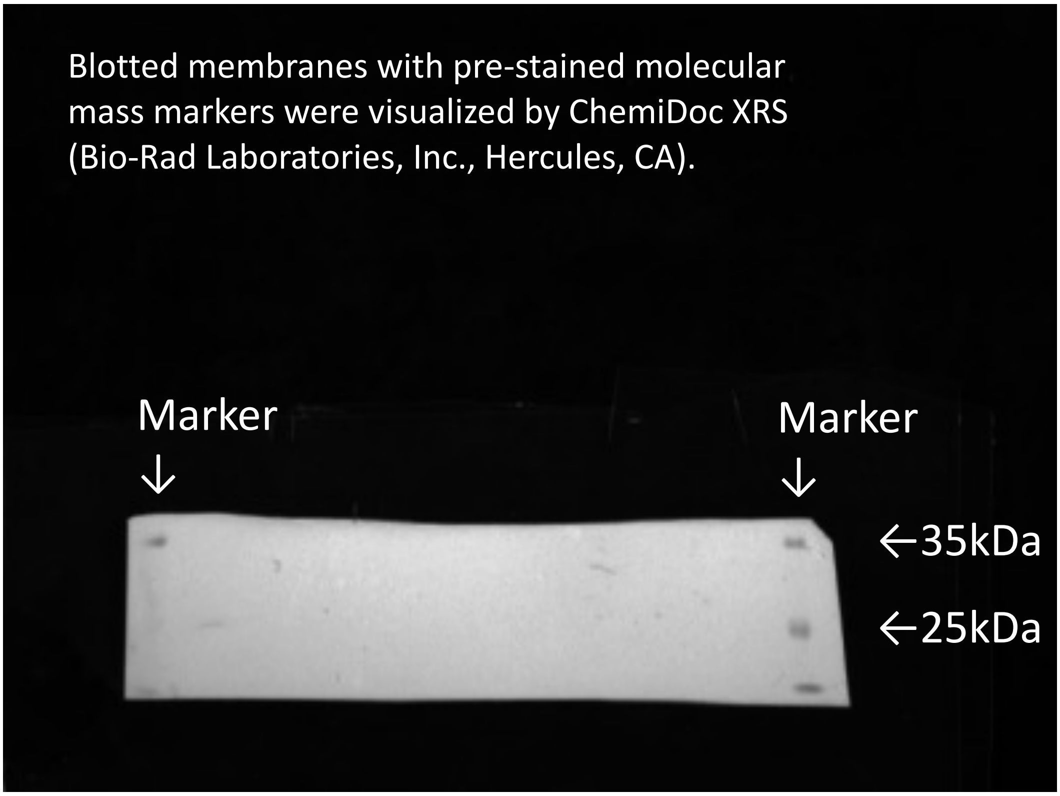

Figure 5a, bottom panel

WB: anti-AAK1 Ab

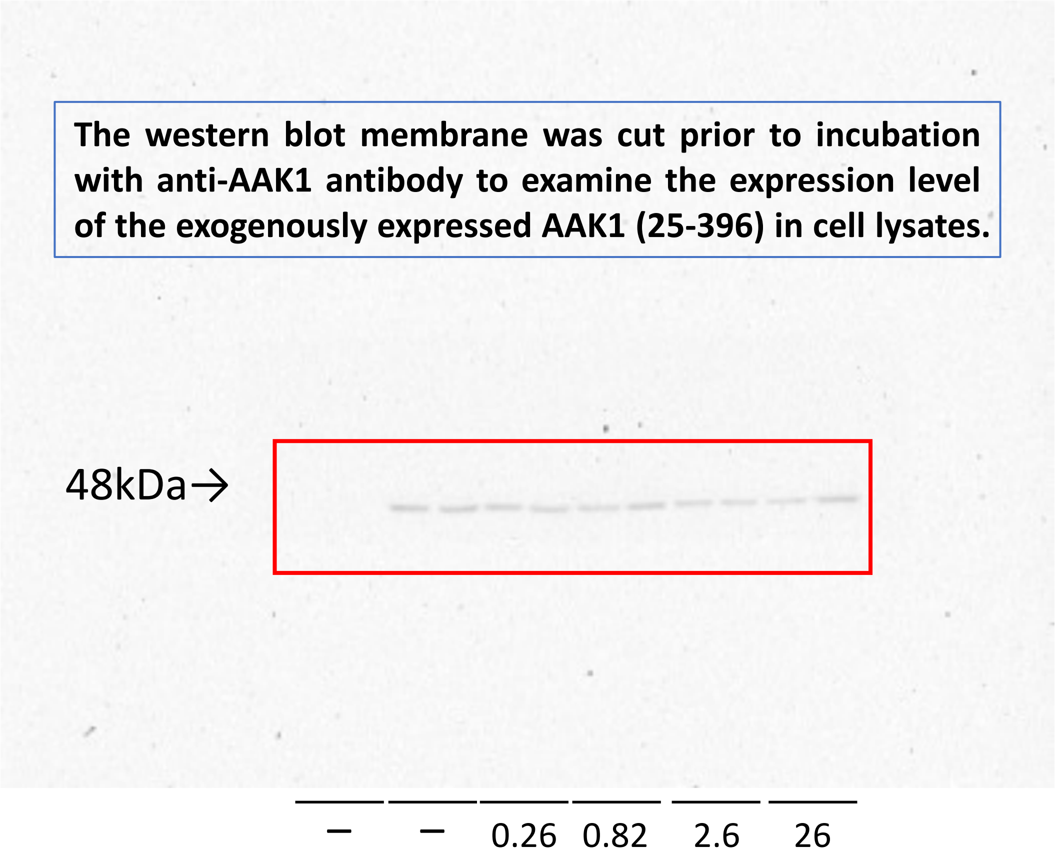

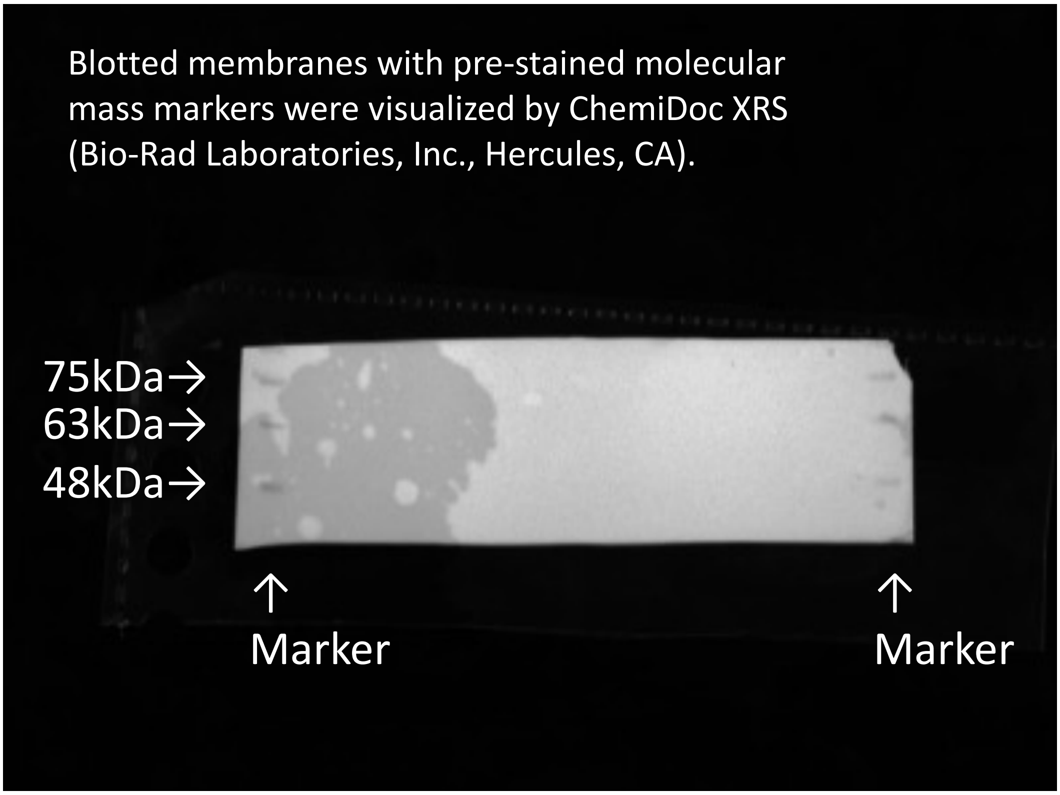

Supplemental Information S3, insert, upper panel

WB: anti-pAP2M1(pThr156) Ab (X-ray film)

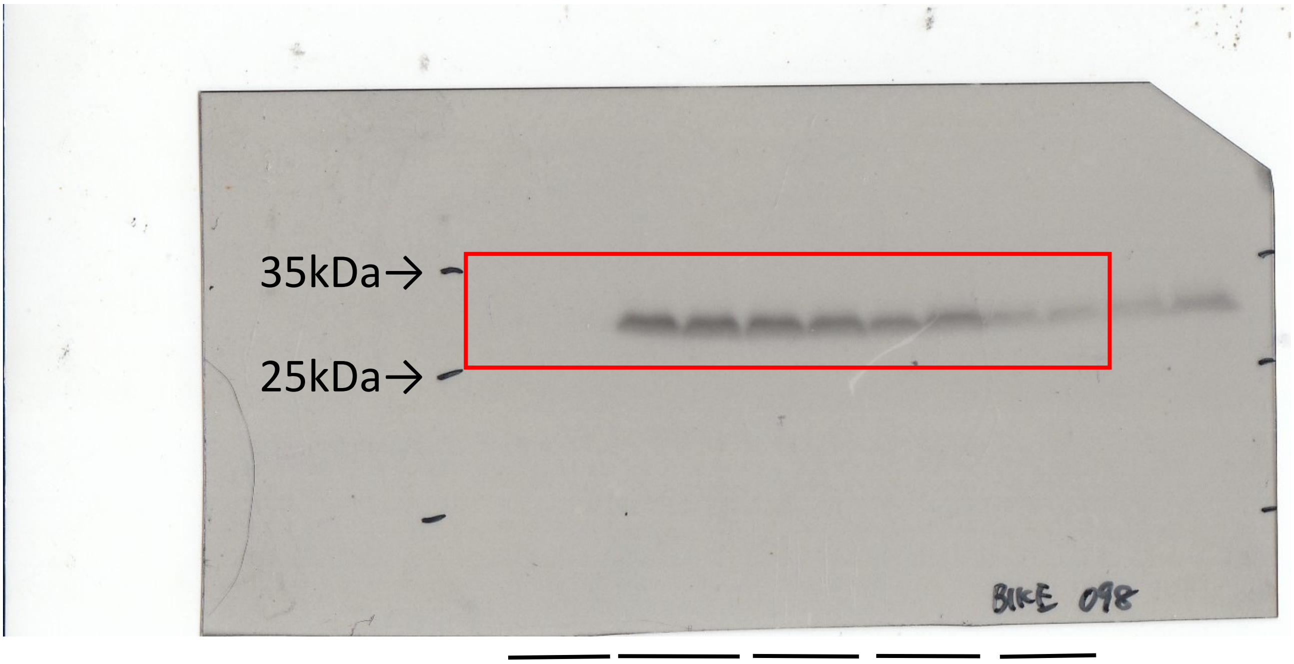

GST-AP2μ2(145-162)	(+)	(+)	(+)	(+)	(+)
BIKE (2-396)	(-)	(+)	(+)	(+)	(+)
TIM-098a(μM)	(-)	(-)	0.26	2.6	26

The western blot membrane was cut prior to incubation with anti-pAP2μ2M1 antibody to examine the phosphorylation level of the substrate protein (GST-AP2μ2 145-162) by BIKE (2-396).

Supplemental Information S3, insert, middle panel

WB: anti-pAP2M1(pThr156) Ab （X-ray film）

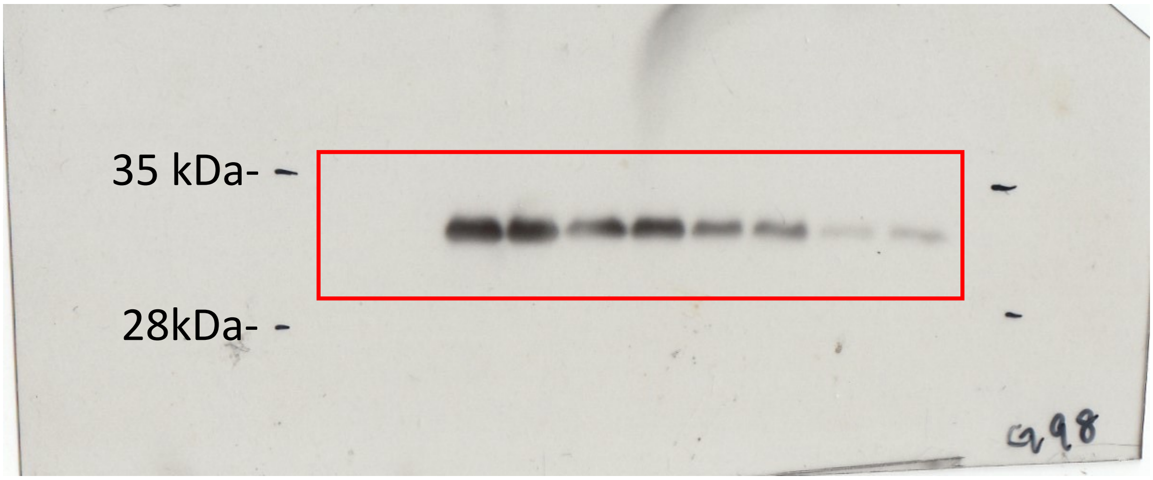

GST-AP2µ2(145-162)	(+)	(+)	(+)	(+)	(+)
GAK (2-370)	(-)	(+)	(+)	(+)	(+)
TIM-098a(µM)	(-)	(-)	0.26	2.6	26

The western blot membrane was cut prior to incubation with anti-pAP2µ2M1 antibody to examine the phosphorylation level of the substrate protein (GST-AP2µ2 145-162) by GAK (2-370).

Supplemental Information S3, insert, bottom panel

WB: anti-pAP2M1(pThr156) Ab (X-ray film)

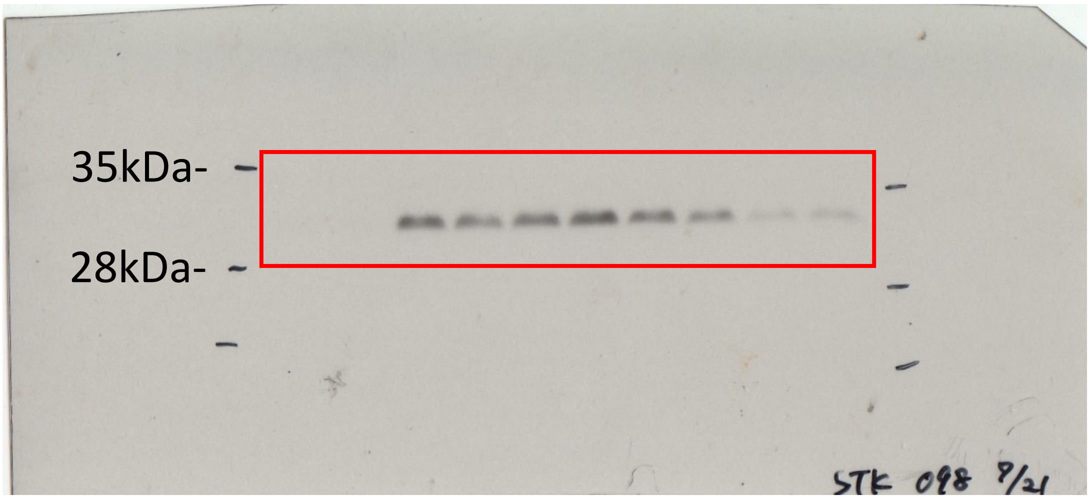

GST-AP2μ2(145-162)	(+)	(+)	(+)	(+)	(+)
STK16 (2-306)	(-)	(+)	(+)	(+)	(+)
TIM-098a (μM)	(-)	(-)	0.26	2.6	26

The western blot membrane was cut prior to incubation with anti-pAP2μ2M1 antibody to examine the phosphorylation level of the substrate protein (GST-AP2μ2 145-162) by STK16 (2-306).
